# Using Genomic Sequencing for Classical Genetics in *E. coli* K12

**DOI:** 10.1371/journal.pone.0016717

**Published:** 2011-02-25

**Authors:** Eric Lyons, Michael Freeling, Sydney Kustu, William Inwood

**Affiliations:** Department of Plant and Microbial Biology, University of California, Berkeley, California, United States of America; University of Missouri-Kansas City, United States of America

## Abstract

We here develop computational methods to facilitate use of 454 whole genome shotgun sequencing to identify mutations in *Escherichia coli* K12. We had Roche sequence eight related strains derived as spontaneous mutants in a background without a whole genome sequence. They provided difference tables based on assembling each genome to reference strain *E. coli* MG1655 (NC_000913). Due to the evolutionary distance to MG1655, these contained a large number of both false negatives and positives. By manual analysis of the dataset, we detected all the known mutations (24 at nine locations) and identified and genetically confirmed new mutations necessary and sufficient for the phenotypes we had selected in four strains. We then had Roche assemble contigs *de novo*, which we further assembled to full-length pseudomolecules based on synteny with MG1655. This hybrid method facilitated detection of insertion mutations and allowed annotation from MG1655. After removing one genome with less than the optimal 20- to 30-fold sequence coverage, we identified 544 putative polymorphisms that included all of the known and selected mutations apart from insertions. Finally, we detected seven new mutations in a total of only 41 candidates by comparing single genomes to composite data for the remaining six and using a ranking system to penalize homopolymer sequencing and misassembly errors. An additional benefit of the analysis is a table of differences between MG1655 and a physiologically robust *E. coli* wild-type strain NCM3722. Both projects were greatly facilitated by use of comparative genomics tools in the CoGe software package (http://genomevolution.org/).

## Introduction

Classical genetic studies often involve selection or screening for new mutations [1], [2]. Traditionally, these mutations have been identified by first mapping them to a small region of the genome and later, as methods became available, determining their sequence changes. In this study we develop computational tools to identify spontaneous mutations in *E. coli* K12 by deep sequencing of its genome.

We contracted with Roche to obtain 454 Titanium FLX shotgun sequences (unpaired) of eight related *E. coli* genomes and compare them to the known genome sequence of MG1655. We then analyzed this data manually and computationally. Our goal was to carry out the computational analysis rapidly, reliably, and in a way that would maximize its usefulness to others under similar circumstances.

Our example centered on the recently described Rut pathway for pyrimidine ring degradation [3]. The transporter and six enzymes of this pathway allow *E. coli* K12 to use pyrimidines as sole nitrogen source at low (19–22°C) but not high temperatures (37°C). To learn something about the effect of temperature, we selected strains that gained the ability to grow on pyrimidines at 37°C. These could not be obtained spontaneously in a wild-type background but were obtained from a strain with elevated transcription of the *rut* operon [*ntrB*(Con), also called *glnL*(Con)]. In addition, we explored the function of the RutE product by selecting spontaneous suppressors of a null allele in *rutE*. A null allele in any *rut* gene results in failure to grow on pyrimidine bases as the sole nitrogen source, even at room temperature, and the *rutE* suppressors restored growth. They could be obtained in an *ntrB*(Con) *ΔrutE* strain, but not in a *ΔrutE* strain.

## Materials and Methods

### Strains and genetic methods

We isolated strains in the background of *E. coli* strain NCM3722 [4] and performed phage P1-mediated transductions as described [3], [5]. All lesions were introduced by transduction, site-directed mutagenesis, or selection of spontaneous mutations. Transductants were purified twice to eliminate phage before they were tested for growth on uridine as the sole nitrogen source. Strains NCM4930, 4931, 4932, 4909, and 4910 were constructed by introducing a lesion in *pdxY*, *anmK*, *lhr*, *ygiC*, or *ygiL*, respectively, into strain NCM3722. Selection was for kanamycin resistance. The lesions were introduced from parental strains JW1628-1, JW1632-1, JW3006-2, and JW3011-2, respectively, [6], which were obtained from the *E. coli* Genetic Stock Center. Spontaneous mutations affecting the Rut pathway were isolated as described in the Introduction [3] and mutations restoring growth at low NH_3_ to strains with inactive forms of the ammonium channel, AmtB, were isolated as described [5].

### 454 sequencing and assembly

We contracted sequencing and assembly to Roche (454 Life Sciences, Branford, CT). We sent 8 to 14.5 µg total DNA at a concentration of 0.8 to 1.5 µg/µl. Roche performed sequencing using 454 GS FLX Titanium series chemistry. They performed both reference assembly by mapping to strain MG1655 (NC_000913) and *de novo* assembly using their proprietary Newbler software. Sequence was not submitted to Genbank for strain NCM3722, its derivatives, and the novel F plasmid because sequence data was not at sufficient coverage to meet minimum Genbank requirements.

### Dideoxy sequencing to confirm 454 predicted mutations

Fragments were amplified by PCR and sequenced using the ABI Prism Big Dye Terminator system v3.1 (Applied Biosystems, Carlsbad, CA).

### Manual Analysis of Contig Breaks

To assemble the virtual genome of NCM3722 we analyzed contigs assembled *de novo* by Roche for strains NCM4139, NCM4287, NCM44299 and NCM4370. We ordered the contigs that carried unique sequence by synteny to MG1655 using Blastn. We then determined the identity of the repetitive elements that caused breaks between contigs by looking at raw sequence reads carrying sequence immediately adjacent to the breaks. In some cases this confirmed that a break was caused by the same repetitive element present in MG1655 at that location and in other instances indicated that it was caused by a different element.

### Assembly of contigs into pseudomolecules by the syntenic path assembly algorithm

The syntenic path assembly algorithm is a four step process to aid assembly of contigs into higher order genomic architecture. 1) It identifies all homologous sequences between a collection of contigs that have been assembled *de novo* and a fully assembled reference genome. 2) It infers synteny between a contig and the reference genome by identifying a collinear series of homologous sequences. 3) It orders and orients the contigs based on their inferred synteny to the reference genome, e.g. their syntenic path along the reference genome. 4) It stitches the contigs together according to their syntenic path. We implemented this algorithm as part of CoGe's [7] SynMap tool. SynMap is a web-based tool that allows researchers to specify two genomes, identify similar sequences [either total DNA or coding sequence (CDS)] using blastn or tblastx [8], infer synteny by collinear arrangements of homologous genes using DAGChainer [9], and display the results in an interactive and informatively colored dotplot. Our data and parameters were: CDS sequences of the reference genome, MG1655 (NC_000913); genomic sequence of contigs assembled *de novo* by Roche using Newbler; blastn with default parameters; e-value cutoff 0.0001; DAGChainer options −g 5 −D 20 −A 5. The syntenic path algorithm is added as an option to SynMap and will order and arrange contigs for display. When selected, a link will be provided to print out the syntenic path assembly of the contigs using 100 nucleotides (100 Ns) to join them.

### Annotation

To predict protein coding gene models in the newly sequenced, assembled genomes we used Prodigal [10] with default parameters. We then used SynMap to identify syntenic gene pairs between each assembled genome and the reference genome and to transpose the annotation from the reference genome. To predict tRNA genes we used tRNA-scan [11] with the “-B” option for bacterial tRNAs. We annotated them according to their acceptor codon and coding amino acid.

### Identification and classification of polymorphisms

To detect polymorphisms we first generated a multiple alignment of the eight genomes from 454 sequencing to the reference genome of strain MG1655, using Mauve's progressive alignment algorithm [12] with default parameters. We chose Mauve for its ability to generate correct alignments of conserved regions even when they are out of order, because our genomes, which were arranged linearly, did not necessarily have exactly the same start position, nor did they have the same start position as the reference genome. The start position depends on the processivity of the *de novo* assembly algorithm and on how the contigs matching the ends of the reference genome were arranged. We wrote a PERL program, PolyMFind, to parse this multiple genome alignment and scan for aligned positions with a SNP, a gap, or an “N” (nucleotide inserted between contigs to stitch them together). These were tabulated for their length and checked against the annotated genomes to determine whether the polymorphism fell within a gene. If so, its position was checked to determine whether it caused a synonymous or non-synonymous change in a codon. In the latter case, the amino acid change was recorded.

### Sources of error and scoring (reliability ranking) of polymorphisms

To prioritize polymorphisms for subsequent evaluation, we employed a simple false positive scoring heuristic for homopolymer errors and misassembly errors. Sequences generated by 454 have errors associated with homopolymers, with error rate increasing with homopolymer length. We assigned a score for homopolymers of length ≥5 that was equal to their length. These constituted 75% of total homopolymer errors in the eight strains and 90% of those in the seven strains with highest sequence coverage (see Results). To assign homopolymer sequencing errors we first considered each putative polymorphism that was a gap or insertion of one nucleotide to be a homopolymer sequencing error. For gaps or insertions of one nucleotide that were identified in multiple whole genomes, the type (missing or extra nucleotide) and length of the homopolymer were assessed based on a majority wins rule. For example, if seven of eight genomes had 5 “A”s, and the eighth genome had 6 “A”s, the putative polymorphism was assigned to the eighth genome and counted as an extra nucleotide in a homopolymer of length 5. The total number of homopolymers of a given length was tabulated for all eight genomes and for the seven genomes with highest fold sequencing coverage. The minimum length for a homopolymer was 1 and the maximum was 9. To calculate the percent homopolymer sequencing error, the number of gaps or insertions of one nucleotide was divided by the total number of homopolymers of that length (see Results).

Short repeated sequences are difficult for assembly programs to process. The error due to misassembly of such sequences into contigs can be estimated by counting the number of polymorphisms within a single gene in a single genome. One can then partition neighboring putative polymorphisms for further evaluation based on the assumption that most of a bacterial genome is constituted by coding sequence. We gave a false positive score for misassembly errors equal to the number of polymorphisms seen within a single gene for a single strain. For example, if one strain had three polymorphisms in a gene, each of these polymorphisms received a misassembly score of 3. In cases where misassembly errors occur outside annotated genes, the tight clustering of polymorphisms that is generated often allows their quick visual identification.

*De novo* assembly of unpaired sequencing reads yields contig breaks at repeat sequences that are longer than the sequencing read, e.g. transposable elements, rRNA operons, and tRNA clusters. Synmap joined neighboring contigs using 100 nucleotides (Ns). Though the presence of these joints was recorded in the multiple genome alignment, no false positive score was assigned. Contig breaks were also recorded for individual strains to help identify new mutations caused by movement of transposable elements and distinguish them from pre-existing occurrences of such elements.

### Assessment of polymorphisms

Even after we developed and implemented a set of criteria to reduce the number of false positives, there were a number of putative polymorphisms to consider. To facilitate further analysis we displayed the output from polymorphism detection as an interactive webpage that permits sorting the results and hiding or showing particular information. It also has links to various comparative genomics tools in CoGe (http://genomevolution.org/) that allow data extraction and quick sequence comparisons at various levels of resolution. These tools facilitate identification of residual homopolymer sequencing and misassembly errors and analyses of contig breaks. The tables and a tarball for the data can be downloaded from http://genomevolution.org/paper_supp_data/8-Ecoli-genomes-2010/

## Results

### Manual analysis of sequence assembled to a non-parental reference genome

From the eight DNA samples sent to Roche (Table 1), we obtained approximately 9.45×10^8^ nt of sequence from 2.34×10^6^ reads, with an average read length between 373 and 420 nt per genome (Table 2). Roche aligned sequence reads for the eight strains against the sequence of the reference strain *E. coli* K12 MG1655 (NC_000913). From these alignments they generated contigs and determined differences between the consensus sequence for each strain and the reference sequence. There were 350–400 differences per strain, which they provided in text and Excel files that included an indication of how often each difference occurred in the sequence reads. Among the differences we found the single nucleotide polymorphisms (SNPs) known to be present in the eight strains because they were present in their parental strains. However, as discussed below, we did not find parental insertion and deletion mutations.

**Table 1:** Strain Table.

Strain	Selection condition	Genotype	Type of Lesion^a^	Reference
NCM4139	Growth on	*ntrB*(Con)^c^ (*ntrB* ^A129T^)	SNP	Kim 2010
	uridine at 37°C	p*lon*::IS186^d^	**insertion**	
		*nemR* ^G141S^	**SNP**	
		intergenic (between *agaA* and *agaS*)	**SNP**	
NCM4287	Suppressor of	*amtB* ^G393A,L394A^ c	SNP, SNP, SNP	Inwood 2009
	*amtB* ^G393A,L394A^ e	*tesB*::kan^c^	insertion	
		*amtB* ^K206K^ c	SNP	
		*chpS* ^S31G^	**SNP**	
NCM4299	Suppressor of	*ntrB*(Con)^c^ (*ntrB* ^A129T^)	SNP	Kim 2010
	*ΔrutE*	*ΔrutE* c	deletion	
		*ΔmioC* c	deletion	
		*nemR* ^1–66, fs^	**fs**	
NCM4300	Suppressor of	*ntrB*(Con)^c^ (*ntrB* ^A129T^)	SNP	Kim 2010
	*ΔrutE*	*ΔrutE* c	deletion	
		*ΔmioC* c	deletion	
		p*nemR* f	**SNP**	
NCM4370	Suppressor of	*amtB* ^L394A^ c	SNP, SNP	Inwood 2009
	*amtB* ^L394Ae^	*tesB*::kan^c^	insertion	
NCM4384	Growth on	*ntrB*(Con)^c^ (*ntrB* ^A129T^)	SNP	Kim 2010
	uridine^b^ at 37°C	*plon*::IS186^c^	**insertion**	
		*ΔmioC*	**deletion**	
		*sroG* (C3182707T)^g^	**SNP**	
NCM4401	Suppressor of	*amtB* ^fs^ c	SNP, SNP, SNP, fs	Inwood 2009
	*amtB* ^fs^ e	*tesB*::kan^c^	insertion	
		*amtB* ^K206K^ c	SNP	
		*ntrB* ^D176E^ h	**SNP**	
NCM4781	Suppressor of	*hflB* ^G361S^ c	SNP	Inwood 2009
	cold sensitivity of	*yehD* ^G118D^	**SNP**	
	*hflB* ^G361S^			

a Lesions identified in this work are bold.

b Uridine as sole nitrogen source, glycerol as carbon source.

c Parental lesion.

d Insertion in the *lon* promoter. The orientation of *insL* in IS186 is indicated relative to the numbering system of MG1655.

e Growth on 0.5 mM NH_4_Cl as nitrogen source at pH 5.5. Glucose was the carbon source.

f Lesion in the *nemR* promoter.

g Lesion disrupts a bp in stem of the *sroG* riboswitch; hereafter designated *sroG* ^m1^.

h Also called *glnL* in *E. coli*.

**Table 2:** Raw data provided by Roche and predicted coding sequences and tRNAs.

Strain	NCM4139	NCM4287	NCM4299	NCM4300	NCM4370	NCM4384	NCM4401	NCM4781
Reads	5.55×10^5^	3.24×10^5^	3.20×10^5^	2.29×10^5^	3.94×10^5^	2.14×10^5^	1.93×10^5^	1.11×10^5^
Bases	23.3×10^7^	13.3×10^7^	12.9×10^7^	9.2×10^7^	14.7×10^7^	8.7×10^7^	7.9×10^7^	4.5×10^7^
Read Length	420	410	403	401	373	406	409	405
Contigs	138	112	127	127	144	117	132	266
Contig Bases	4.661×10^6^	4.659×10^6^	4.643×10^6^	4.643×10^6^	4.655×10^6^	4.643×10^6^	4.654×10^6^	4.640×10^6^
Fold Coverage	51	30	29	20	33	19	18	10
Coding^a^ Sequences (CDS)	4196	4222	4208	4221	4210	4224	4244	4213
tRNA^b^	46	47	45	48	49	45	46	43

a Predicted using Prodigal; see Fig. 1.

b Predicted using t-RNA scan.

The eight strains sequenced were isolated as spontaneous mutants able to use pyrimidines at the normally non-permissive temperature of 37°C, or as spontaneous suppressors of *rutE*, *amtB*, or *hflB* lesions. To identify the new mutations that had been selected in these strains (see Introduction and Materials and Methods; [3], [5], [13]), we made pair-wise comparisons in the text files at each location where a difference was noted. In these files individual sequence reads were shown aligned to MG1655 sequence. In this way we found: *nemR* mutations in NCM 4139 and NCM4299; a mutation in the *nemR* promoter in NCM4300; a *chpS* mutation in NCM4287; an *sroG* mutation in NCM4384; and an *ntrB* (*glnL*) mutation in NCM4401 (Table 1). We also found a mutation in non-coding sequence between *agaA* and *agaS* in NCM4139. In NCM4781 we found a *yehD* mutation, despite the fact that sequence coverage in this strain was low (see below), which led to more than double the number of false positives seen in other strains and a complete lack of sequence coverage in some regions of the genome. Though sequence coverage was adequate, we found no new mutation in NCM4370 for reasons described later.

Over 90% of the differences in the Roche files were common to all eight strains and hence were false positive candidates for mutations. Many were due to assembly errors or sequencing variability. Errors often resulted from incorrect assembly of repetitive elements or larger near-repetitive elements such as tRNA clusters and the DLP12, RAC, and QIN cryptic prophages. This occurred because the decision structure of the Newbler assembly program could not determine where to place repetitive elements relative to the reference sequence (MG1655). Most of the remaining differences were due to polymorphisms between our strain background and strain MG1655. We did not send the parent strain NCM3722 itself to Roche for sequencing.

Differences between strain NCM3722, and hence common to all of our strains, and MG1655 did not appear in the data tables. For example, our strains all contain an F plasmid, whereas MG1655 does not (Table S1). In order to determine the location of the various IS elements common to our eight strains, we used the known terminal sequence for each element to search raw sequence data. In this manner we determined the locations for IS1, IS2, IS3, IS4, IS5, IS30, IS150, and IS186. We found 14 differences in their location between NCM3722 and MG1655 (Table S1). Using the same method, we found a lambda prophage and a TN1000 element and were led to 7 kb of new sequence, including six intact genes, in NCM3722 but not MG1655. We were also able to identify three other mutations known to be present in our parental strains that were insertion or deletion mutations (*tesB*::kan, *ΔrutE* and an 11 kb deletion of *mioC* through *rbsB*, which we refer to as *ΔmioC*; Table 1) [3], [5]. Most important, we identified the IS186 element at a new location within the *lon* promoter of two of our strains, NCM4139 and NCM4384 (Table 1). It represented a candidate mutation to explain the phenotypes of these strains.

### Genetic analysis of candidate mutations

P1-mediated transduction was used to determine whether the new mutations identified by manual analysis caused the phenotypes we had selected. We have shown previously that a *nemR* mutation is sufficient to suppress the absence of RutE [3] and hence that the *nemR* and *nemR* promoter mutations present in strains NCM4299 and 4300, respectively, are sufficient to account for their phenotypes. In this study we wanted to determine if the *nemR* and *sroG* lesions identified in NCM4139 and NCM4384, respectively, were necessary and sufficient for growth on pyrimidines as the sole nitrogen source at 37°C. To determine whether they were necessary we first used phage P1-mediated transduction to introduce insertion/deletions in markers that were linked to these loci and conferred kanamycin resistance. We then scored drug-resistant transductants for retention of the ability to grow on pyrimidines at high temperature. The linked markers we used for *nemR* were *pdxY*, *anmK*, and *lhr*, all of which appeared to be dispensable for growth on minimal medium (http://biocyc.org/ecocyc/index.shtml). For *sroG*, the linked markers were *ygiC* and *ygiL*, which likewise appeared to be dispensable for growth on minimal medium. In each case, at least some of the transductants lost the ability to grow on pyrimidines at 37°C: the closer the marker was to *nemR* or *sroG*, the larger was the number of transductants that lost this ability (Table 3). Transductants that lost growth also lost the lesion in *nemR* or *sroG*, whereas transductants that retained growth retained the lesion. These results indicate that the *nemR* or *sroG* lesion was required for growth on pyrimidines at high temperature. The insertion of IS186 that was present in the *lon* promoter in both NCM4139 and NCM4384 was retained in all the transductants we examined from all crosses, and hence the results indicated that the *lon* promoter mutation alone was not sufficient to confer growth on uridine at 37°C. Likewise the *mioC* deletion in strain NCM4139 was retained and hence was not sufficient to confer growth even in the presence of the *lon* promoter mutation, and vice versa.

**Table 3:** The *nemR* or *sroG* lesion is necessary and sufficient for growth on uridine at 37°C.

Paternal	Maternal	Distance in kilobases between marker and mutation of interest	Transductants that grow at 37°C/Total transductants examined	Relevant phenotypes and genotypes of transductants
NCM4930	NCM4139	11.5	4/10	NCM4933 (+)^a^
*pdxY*::kan	*nemR*			*nemR* ^G141S^ p*lon*::IS186
				NCM4938 (−)^b^
				*nemR* ^+^ p*lon*::IS186
NCM4931	NCM4139	6.9	2/10	NCM4934 (+)
*anmK*::kan	*nemR*			*nemR* ^G141S^ p*lon*::IS186
				NCM4939 (−)
				*nemR* ^+^ p*lon*::IS186
NCM4932	NCM4139	2.6	1/10	NCM4935 (+)
*lhr*::kan	*nemR*			*nemR* ^G141S^ p*lon*::IS186
				NCM4940 (−)
				*nemR* ^+^ p*lon*::IS186
NCM4909	NCM4384	2.3	2/122	NCM4936 and NCM4937 (+)
*ygiC*::kan	*sroG*			*sroG* ^m1^ p*lon*::IS186 *ΔmioC*
				NCM4965 and NCM4966 (−)
				*sroG* ^+^ p*lon*::IS186 *ΔmioC*
NMC4910	NCM4384	0.7	0/122	NCM4967 and NCM4968 (−)
*ygiL*::kan	*sroG*			*sroG* ^+^ p*lon*::IS186 *ΔmioC*
NCM4933	NCM3876	11.5	8/12	NCM4943 (+)
*pdxY*::kan	*ntrB*(Con)			*nemR* ^G141S^ p*lon* ^+^
*nemR* ^G141S^				
NCM4934	NCM3876	6.9	7/12	NCM4944 (+)
*anmK*:kan	*ntrB*(Con)			*nemR* ^G141S^ p*lon* ^+^
*nemR* ^G141S^				
NCM4935	NCM3876	2.6	12/12	NCM4945 (+)
*lhr*::kan	*ntrB*(Con)			*nemR* ^G141S^ p*lon* ^+^
*nemR* ^G141S^				
NCM4936	NCM3876	2.3	8/8	NCM4948 and NCM4949 (+)
*ygiC*::kan	*ntrB*(Con)			*sroG* ^m1^ p*lon*+ *mioC* ^+^
*sroG* ^m1^				

a Grows on uridine at 37°C.

b Fails to grow on uridine at 37°C.

We assessed whether the *nemR* lesion present in NCM4139 and the *sroG* lesion in NCM4384 were sufficient for growth on uridine at 37°C. To do this, we grew phage on transductants that carried kanamycin insertions in markers linked to *nemR* or *sroG* and also retained the *nemR* or *sroG* lesion. We used this phage to transduce an *ntrB*(Con) strain to drug-resistance on enriched medium at 37°C and scored transductants for acquisition of the ability to grow on uridine as the sole nitrogen source at 37°C. In all cases we obtained transductants that had acquired this ability: their number reflected the closeness of the marker to *nemR* or *sroG* (Table 3). We showed that such transductants carried the *nemR* or *sroG* lesion. Transductants that had not acquired the ability to grow on pyrimidines at high temperature remained wild-type at *nemR* or *sroG*. As expected, none of the transductants had acquired either the IS186 insertion in the *lon* promoter or the deletion around *mioC*. The results indicated that the *nemR* or *sroG* lesion was sufficient for growth on pyrmidines as sole nitrogen source at 37°C. However, we noted that none of the transductants grew as robustly as NCM4139 and NCM4384 or their derivatives carrying markers linked to the *nemR* or *sroG* loci.

A third set of transductional crosses indicated that robust growth on pyrimidines at 37°C required the *lon* promoter mutation in addition to a *nemR* or *sroG* mutation. For these crosses we used phage grown on NCM4139 or NCM4384 to transduce an *ntrB*(Con) strain directly to growth on uridine at 37°C. In each case there were only a few transductants (∼50) and these took at least a week to appear. After purification, the transductants grew as well as the donors. Some failed to grow at all and we presume these were contaminants from the background, although we cannot exclude that they were recombinants or spontaneous mutants that carried the *lon* promoter mutation. The two transductants that we checked from the cross with NCM4139 as the donor carried its *nemR* ^G141S^ lesion, which we presume was inherited by recombination (Table 4). They also carried an IS186 insertion in the *lon* promoter. Although the insertion in the transductants was at the same position as in the donor, in one transductant it was in the opposite orientation. This provided evidence that the insertion was acquired spontaneously. Neither transductant carried the *mioC* deletion. The results showed that the combination of the *nemR* and *lon* promoter mutations conferred robust growth on uridine at 37°C. It will be of interest to understand the basis for the synergism between these mutations.

**Table 4:** Robust growth on uridine at 37°C requires the *lon* promoter mutation.

Paternal	Maternal	Transductants analyzed^a^	*nemR*/*sroG* genotype^b^	*lon*/*mioC* genotype
NCM4139	NCM3876	NCM4951	*nemR* ^G141S^	*plon*::IS186^c^
*nemR* ^G141S^	*ntrB*(Con)	NCM4958	*nemR* ^G141S^	*plon*::IS186^c^
*lon*::IS186^c^				
NCM4384	NCM3876	NCM4952	*sroG* ^+^ p*nemR* d	*plon*::IS186^c^
*sroG* ^m1^	*ntrB*(Con)			*mioC* ^+^
*plon*::IS186^c^		NCM4960	*sroG* ^+^ rbs*nemR* e	*plon*::IS186^c^
				*mioC* ^+^
		NCM4961	*sroG* ^+^ p*nemR* d	*plon*::IS186^c^
				*mioC* ^+^
		NCM4962	*sroG* ^m2^ *nemR* ^+^	*plon*::IS186^c^
				*mioC* ^+^

a All grew on uridine at 37°C.

b The *sroG* ^m1^ mutation is C3182707T and the *sroG* ^m2^ mutation is A3182644G. Both are in stems of the riboswitch structure and would prevent base pairing.

c The orientation of *insL* in IS186 is indicated relative to the numbering system of MG1655.

d The *nemR* promoter mutations are the same as that in NCM4300. They are predicted to eliminate repression of the *nemRA* operon by NemR [3].

e The mutation is in the ribosome binding site for *nemR*.

Surprisingly, none of the four transductants derived from the cross with NCM4384 as donor carried the parental *sroG* lesion (Table 4). One had a new lesion in *sroG* and the other three had lesions in the promoter or ribosome binding site for *nemR*. All four also carried an IS186 insertion in the *lon* promoter. In two cases it was present in the opposite orientation from that in the parental strain (and in the same orientation as in NCM4139). Hence in these two cases, it must have arisen spontaneously and so neither lesion arose by recombination. In the other two cases it is possible that the insertion was transferred by recombination and only the *nemR* lesion arose spontaneously. As was the case for *nemR*, a *lon* promoter mutation appears to be synergistic with *sroG* in conferring robust growth on uridine at 37°C. The *lon* promoter mutations appear to arise readily by spontaneous transposition of IS186 to a single site, and in either orientation [14]. Moreover, *nemR* and *sroG* appear to be the two primary targets for allowing growth on pyrimidines at 37°C.

Three of the remaining strains sent for sequencing, NCM4287, NCM4370, and NCM4401, contained suppressors of C-terminal lesions that inactivate the ammonium channel AmtB [5], [13]. Though most of the intragenic and extragenic suppressors of the *amtB* lesions had been found previously [5], [13], extragenic lesions in these three strains, which constituted a very small proportion of the total, had not. We identified unique mutations in *chpS* and *ntrB* (*glnL*) in strains NCM4287 and NCM4401, respectively, but failed to identify a candidate lesion in NCM4370. The last strain we had sequenced, NCM4781 contains a suppressor of the cold sensitivity caused by an *hflB* mutation [5]. Although sequence coverage was poor, we identified a candidate lesion in *yehD*. Others in our group are currently evaluating the three candidate lesions for their roles in the phenotypes that were selected (J. A. Hall, unpublished results).

### Computational analysis of sequences assembled *de novo*

Because the manual approach we used to identify new mutations in our strains was labor intensive and because reference genome assembly systematically missed insertion elements, we asked Roche to perform a *de novo* assembly for each of our strains (Fig. 1, Table 2). The *de novo* assembly yielded between 112 and 266 contigs per genome for assembled genome sizes that would range from 4.643×10^6^ to 4.654×10^6^ bp. Coverage was between 10- and 51-fold (Table 2). With unpaired sequencing, repetitive sequences that are longer than the average read length (e.g. prophage, rRNA gene cassettes, transposons) cannot be unambiguously assembled and force *de novo* assembly algorithms to terminate regardless of the fold sequence coverage. In our case *de novo* contig assembly reached saturation by 18-fold coverage (Figure 2), i.e. the total number of contigs did not decline after that. Further fluctuations in their number as fold coverage increased appear to be due to efforts of the Newbler assembly program to distinguish between repetitive elements.

**Figure 1:**
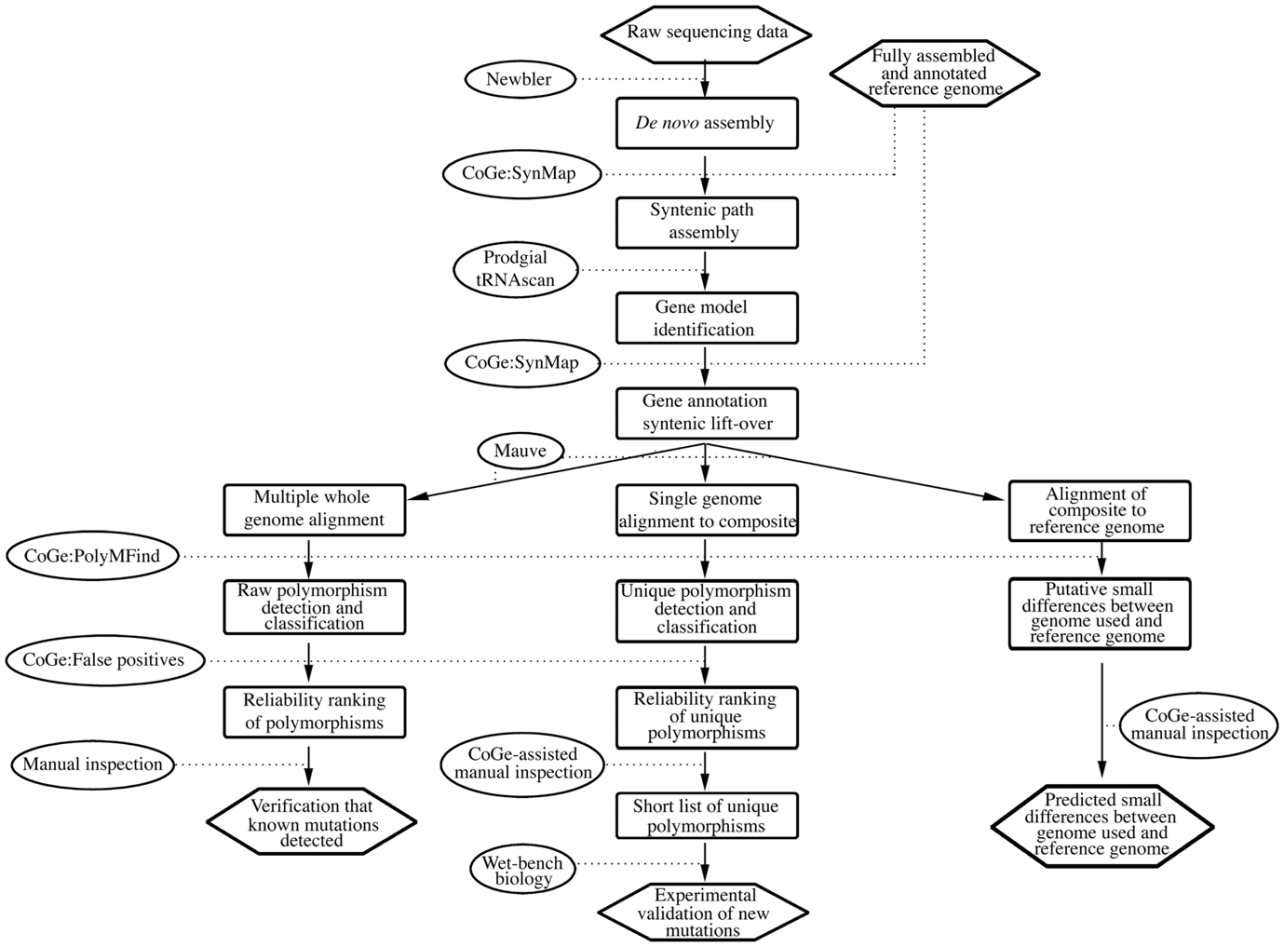
Computational analysis of sequencing data. Hexagons represent initial data sets and final outputs; ovals represent algorithms and other operations; rounded boxes represent data transformations. Note that Mauve produces alignments of multiple genomes and that the logic for construction of a composite sequence is internal to PolyMFind during polymorphism detection. The net effect of these two programs is the comparison of one genome to a composite for the identification of unique polymorphisms.

**Figure 2:**
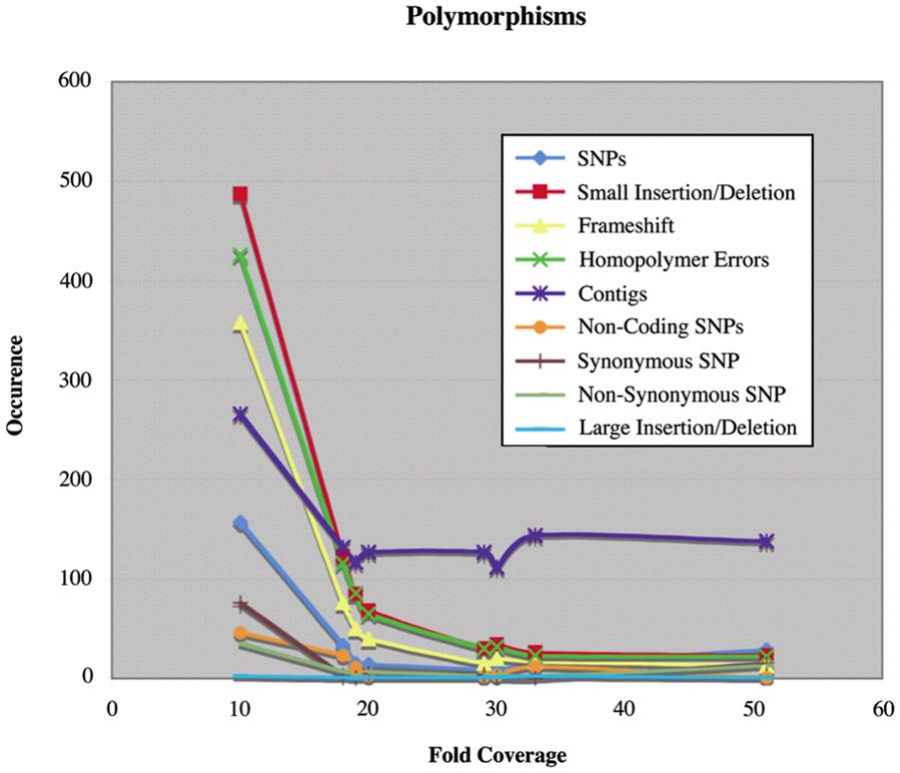
Number of contigs and polymorphisms of various classes and subclasses (y-axis) vs. fold sequence coverage (x-axis). See Table S1.

### Syntenic path assembly

**Figure 3:**
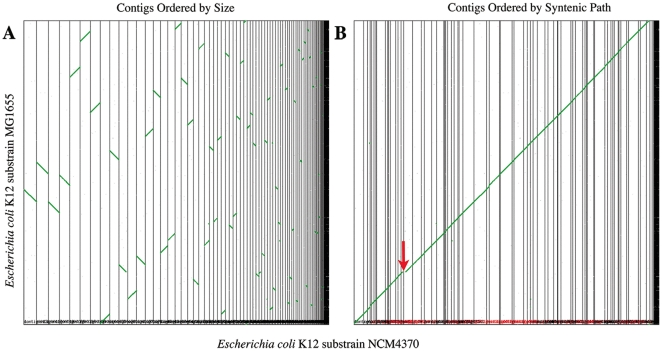
Syntenic dotplots of *de novo* assembled contigs of NCM4370 (x-axis) to fully sequenced and assembled reference genome MG1655. Vertical black lines separate contigs from NCM4370. Gray dots are putative homologous gene pairs. Green dots (which form lines) are collinear sets of homologous gene pairs used to infer synteny. (A) NCM4370 contigs are ordered by size with largest on the left (http://genomevolution.org/r/bjz). The largest contigs are in the region of the terminus of replication, which is known to contain fewer repetitive elements than other regions of the *E. coli* genome [46]. (B) NCM4370 contigs are ordered in the best syntenic path by comparison to reference genome MG1655. Individual contigs may be inverted to ensure that the syntenic path is conserved. Discontinuities in syntenic line are the result of deletions and insertions. The red arrow marks the position of a lambda prophage in NCM4370 (see text). Results can be regenerated at: http://genomevolution.org/r/bjy.

To assemble contigs into full-length pseudomolecules — which we refer to as “stitching” them together — we used syntenic path assembly (see Materials and Methods). The SynMap tool in CoGe's suite of tools implements this algorithm to order contigs and determine their orientation through syntenic comparison with a reference genome, in this case *E. coli* strain MG1655 (Figure 3). This hybrid approach minimizes assembly errors due to insertions in the experimental genome(s) but not the reference genome. An example of such an insertion is the lambda prophage (Fig. 3B, red arrow; Fig. S1), which was missed in the reference genome assembly we used for manual analysis. Syntenic path assembly will miss inversions with endpoints in repetitive regions that give rise to contig breaks [15].

### Genome annotation

After assembling the genomes, we annotated them using Prodigal (see Materials and Methods). Of the 33,738 predicted protein coding genes, 31,431 had a syntenic partner in the reference genome, i.e. 2307 did not. The mean number of predicted protein coding genes was 4217 in each of our eight strains and 4144 in reference strain MG1655 (NCBI reference sequence NC00913; Table 2). The genes in our eight strains that lacked a syntenic partner in MG1655 were checked against other *E. coli* genomes, bacteriophages, and NCBI's non-redundant (NR) sequence database. Some are due to insertions such as prophages (Fig. S1) or extrachromosomal elements such as the F plasmid. Others are due to: Prodigal annotation errors, e.g. split gene models; genes annotated as pseudogenes in MG1655; genes missed by the syntenic gene pairs assignment algorithm.

On average, tRNA-scan predicted 46 tRNAs for each of our eight strains (Table 2). This was significantly lower than the 86 tRNAs annotated in the reference genome, MG1655. Although the genomic distribution of the predicted tRNAs was concordant between the strains we sequenced and MG1655 (Fig. S2), our strains were missing tRNAs that occurred in tandem at a single locus in the reference strain. Repetitive sequences in tandem are problematic for *de novo* assembly algorithms and these tRNAs are almost certainly present in our eight strains, despite the fact that they are not present in contigs. Twenty seven of the 40 missing tRNAs were in clusters that caused contig breaks. Another 11 were in ribosomal RNA clusters, which also caused contig breaks.

### Causes of contig breaks

We further exploited SynMap to determine the causes of contig breaks and search for new mutations due to movement of insertion elements. In the seven strains with the highest sequence coverage (see below) we identified 451 contig breaks — which are called contig joins when identified computationally —an average of 64 per strain (7-strain table at http://genomevolution.org/paper_supp_data/8-Ecoli-genomes-2010/). These contig joins were at 95 locations, of which 47 were in common between all strains. The 47 common contig joins were a subset of the 62 true contig breaks identified in the next section describing the manual assembly of the virtual genome of NCM3722. Six of the remaining true breaks correspond to multiple, closely apposed contig joins (7-strain table). Of the remaining nine true breaks one is at the end of the pseudomolecule representation of a circular chromosome, one involves two IS elements that are separated by only 42 bp, and seven are due to gaps in the pseudomolecule assembly caused by the requirement that a contig carry five contiguous homologous genes to be stitched into the pseudomolecule. This parameter can be modified by changing DAGChainer options (see Materials and Methods; [9]). The gaps, which are 3.0, 2.4, 4.2, 1.6, 5.4, 2.8, and 4.3 kb, can be seen by comparing any one of the seven strains with the MG1655 reference strain using the GEvo link provided in the 7- and 8-strain tables. In most cases such a comparison also allows a correct interpretation of the cause of the gap. Graphical representations of such comparisons, such as the one shown in Fig. S1, are particularly useful.

**Figure 4:**
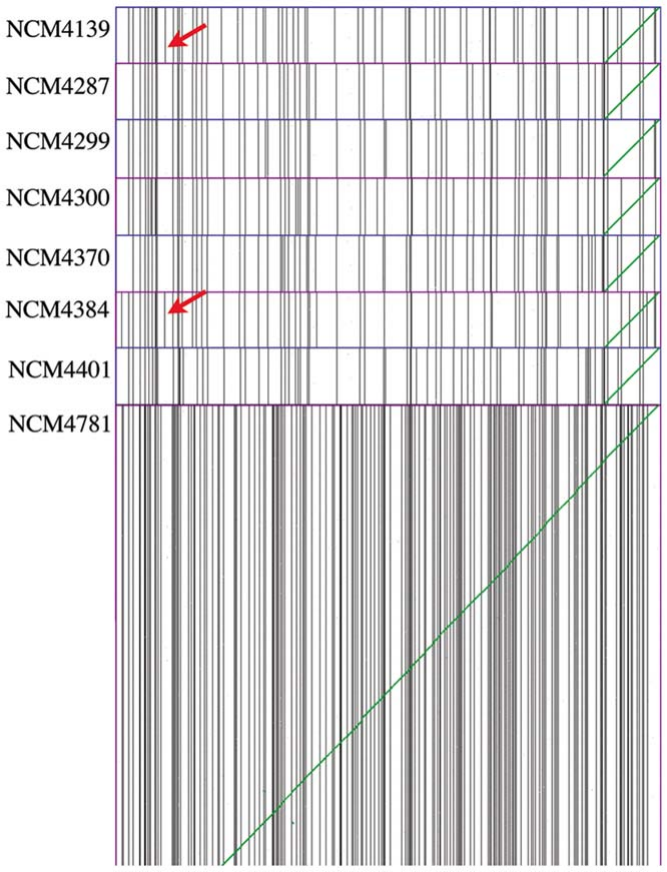
A series of syntenic dotplots between the NCM strains and the reference genome MG1655. Scaffolds of the NCM strains are ordered by their syntenic path to MG1655. Vertical black lines are divisions between contigs and green diagonal lines are syntenic gene pairs. Red arrows show an additional contig break in NCM4139 and NCM4384 caused by a new insertion of IS186 in the promoter for the *lon* gene. The extra breaks in strain NCM4781, which were due to insufficient sequence coverage, are immediately apparent.

Another way to study contig breaks is to align syntenic dotplots of each strain with MG1655 and show the positions of contig joins with vertical lines (Fig. 4). The joins can be visualized by using the interactive tool GEvo in CoGe. Among the 48 contig joins that were present in subsets of the seven strains with best sequence coverage (2 in six strains, 4 in five strains, 7 in four strains, 9 in three strains, 9 in two strains, and 17 in one strain), one in particular was noticeable and is marked with an arrow (Fig. 4). It was present in only two strains (NCM4139 and NCM4384) and was located in the promoter for the *lon* gene. This was the new IS186 insertion (Table 1). Most of the other 47 joins present in only subsets of our strains were clustered and we could assess by using the interactive tools in CoGe that they were caused by slight differences in the positions of contig breaks in rRNAs, tRNA clusters, REP sequences [16], [17], rhs elements [18], [19], and duplicated genes and thus did not represent new mutations in any of our strains.

### Raw polymorphisms

We identified a total of ∼1800 putative polymorphisms among our eight strains, as described in Materials and Methods (Table 5, Table S2, 8-strain table). After subtracting contig breaks (∼600), the remaining polymorphisms consisted of ∼300 SNPs, ∼900 indels smaller than 100 nt, and 9 indels larger than 100 nt. When we analyzed the data by strain, we found that the total number of predicted polymorphisms was inversely related to fold sequence coverage (Fig. 2; Table S2), as was the number of predicted polymorphisms of various classes and subclasses. Major types of polymorphisms considered together (SNPs and small indels) decreased substantially at 20-fold coverage and continued to fall through 30-fold. Between 10- and 20-fold coverage, this number fell 75% and it fell an additional 57% between 20- and 30-fold coverage. It remained relatively constant between 30- and 50-fold-coverage. The slight rise at 50-fold appears to be due to efforts of the Newbler assembly program to assemble repetitive sequences.

**Table 5:** Summary of Polymorphisms.

Strains considered	Total putative polymorphisms	Without contig breaks	Without contig breaks or multiple occurrences
Eight	1779	1164	1022
Seven^a^	995	544	440
Seven after false positive scoring^b^	619	168	120

a Strain NCM4781, which had only 10-fold sequence coverage, was omitted.

b The number of confirmed mutations in the seven strains was 30 without contig breaks and 16 without contig breaks or multiple occurrences.

When NCM4781, which had the lowest fold sequence coverage, was removed from the comparison, the total number of predicted polymorphisms in the remaining seven strains was reduced to ∼1000 and in the absence of contig breaks to ∼550 (Table 5; 7-strain table). Among them were all of the known and newly-identified differences we had found between the seven strains manually, apart from the IS186 insertions in the *lon* promoter (30 total). However, the “true” mutations constituted just 6% of the total putative polymorphisms. There were two major causes of false positives: 1) differences in the consensus sequence of homopolymer regions in different strains due to sequencing errors (homopolymer sequencing errors) and 2) misassembly of locally repeated sequences, particularly tRNAs and small repetitive elements (misassembly errors). Homopolymer sequencing errors declined to their lowest level at 20-fold sequence coverage (Fig. 2).

### False positives and ranking of putative polymorphisms

**Figure 5:**
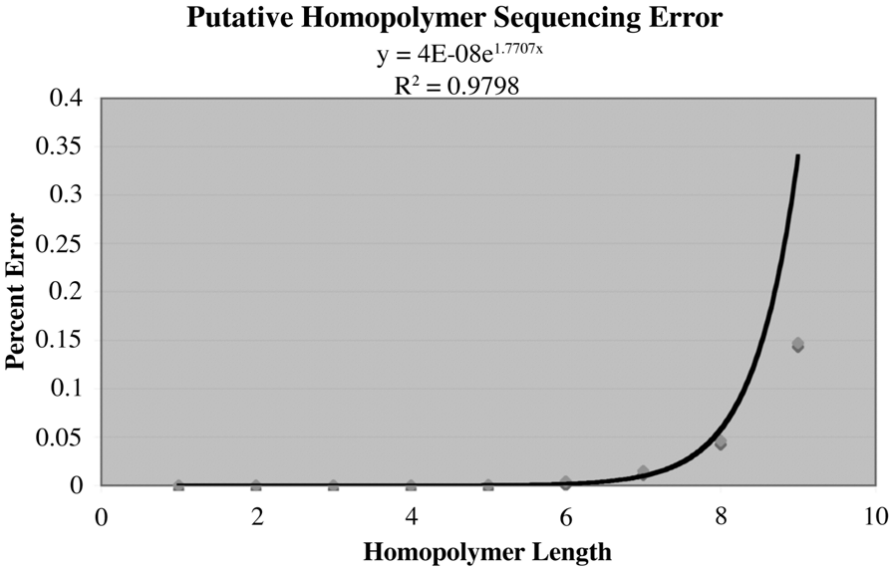
Percent homopolymer sequencing error versus homopolymer length with exponential regression. Data are plotted for the seven strains with highest sequence coverage (see Table S2).

In 454 sequencing, homopolymer errors are known to increase with homopolymer length [20]. For our seven strains with the best sequence coverage, the increase fit very well to the exponential function f(x) = 4×10^−8^ e^1.707x^ with a coefficient of determination (R^2^) of 0.9798 (Fig. 5). Approximately 90% of the total homopolymer errors occurred for lengths ≥5 (Table S3). Hence, we penalized these with scores equal to their length and assigned no penalty to putative polymorphisms in shorter homopolymers.

Small repetitive sequences cause either contig breaks or misassembly errors. We penalized those misassembly errors that gave rise to multiple polymorphisms within a single coding sequence by assigning a score equal to the number of occurrences and compensated for the lack of a more adequate system by annotating tRNAs. Tandemly repeated tRNA genes are one major source of the remaining misassembly errors and their annotation allows the experimentalist to discount them at will.

**Figure 6:**
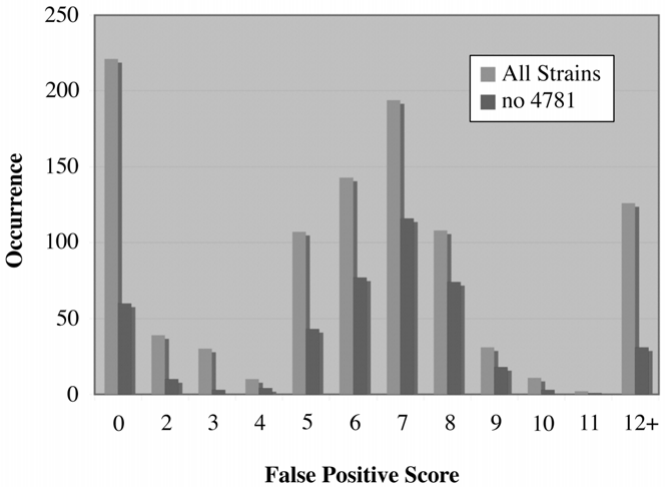
Number of polymorphisms as a function of false positive score. The number of putative polymorphisms was determined for all eight strains and for the seven strains with highest sequence coverage. The number of known and confirmed new mutations in the seven strains was 30 and all had false positive scores ≤5.

After sorting contig breaks to the bottom of the 7-strain table and applying false positive scores to the remaining entries, the 30 mutations present in the seven strains, apart from the IS186 insertions in the *lon* promoter, were found among the 168 putative polymorphisms with lowest false positive scores (≤5) (Fig. S3, Fig. 6, 7-strain table). In other words, the real mutations now constituted 18% of the total. Many of the remaining putative polymorphisms could be eliminated without dideoxy sequencing by using the links in the sorted table. Homopolymer errors were conspicuous in the column “show,” which gives the immediate sequence context of the polymorphism across all strains. Most misassembly errors were due to repeated sequences that were not in genes and these were often clustered. They could be detected using the link to GEvo, which permits high-resolution sequence comparison and facilitates detection of local repeats in the region. Additional polymorphisms due to sequencing errors could be identified by inspecting raw sequence data.

### Unique polymorphisms in individual strains

To facilitate detection of just the new mutations that had been selected in the seven strains with best sequence coverage we identified polymorphisms unique to each strain. These were detected by comparing the sequence of each individual strain to a composite sequence derived from the remaining six. The composite sequence included all positions at which the six strains were invariant. It excluded the small number of positions where known mutations or putative polymorphisms were present in more than one strain. To examine the “single strain” tables, each of which contained approximately 50 entries, we first sorted contig breaks to the top to remove them from consideration and then sorted by false positive score. New mutations in each strain were found among the few candidates with a score <5 (Table S4; single-strain tables). In examining the single strain tables, we noted that 30-fold sequence coverage (NCM4287 NCM4299, and NCM4370) was optimal for detecting new mutations. Lower coverage — 20-fold in NCM4300, NCM4384, and NCM4401 — yielded greater numbers of clustered putative polymorphisms, thereby making it slightly more difficult to detect new mutations.

For strain NCM4139, there were only four candidates with false positive scores of 0 and then the score jumped to 5. The real *nemR* lesion in this strain, a SNP, was among the candidates with a score of 0. In the automated annotation *nemR* is *ydhM*, with the “y” designating a gene of unknown function. Inspection of sequence around the remaining three candidates with a 0 score using CoGe's “Show” function and raw sequence data revealed that the putative *yfaD* polymorphism was due to a sequencing error and hence could be discounted. Likewise, the putative *yiiM* polymorphism was due to an assembly error. The intergenic polymorphism at position 3,206,230, which lay between the *agaA* and *agaS* genes, was confirmed by resequencing. It was the only unselected mutation we confirmed independently. As expected, the *ntrB* (*glnL*) lesion known to be present in NCM4139 did not appear in the table because it was present in three additional strains.

For strain NCM4287, there were four candidate polymorphisms with a false positive score of 0 and two with a score of 2. The score then jumped to 5. Looking at the position of these mutations revealed that four of the candidates with good scores (0 or 2) occurred in pairs (one pair at 2,021,439 and one pair at 4,376,124; Table S4) and provided initial evidence that they were false positives. Inspection of sequence around these lesions indicated that all four were due to homopolymer sequencing errors. The first pair and one member of the second pair were due to an incorrect choice by the global alignment algorithm of where to place a gap caused by a homopolymer sequencing error, a rare occurrence, and the last was below the length cutoff of five that we used to detect homopolymer sequencing errors. The intergenic lesion at position 2,969,777 was also due to a homopolymer sequencing error. The *chpS* lesion appears to be real but we have not yet analyzed it genetically. As expected, the *tesB* lesion in this strain was not detected because it is also found in NCM4370 and NCM4401. Likewise, the *amtB* lesion was not detected because it overlaps one in NCM4370 and the silent lesion in *amtB* was not detected because it was also present in NCM4401.

For strain NCM4299, there were only two candidate polymorphisms with false positive scores equal to 0. The score then jumped to 5. One candidate with a good score was the real *nemR* (*ydhM*) lesion and the other was a sequencing error, as determined by checking the raw data. The *rutE*Δ and the *mioC*Δ known to be present in NCM4299 were not in the table because they were also present in NCM4300, and the *ntrB* (*glnL*) lesion has already been discussed.

For strain NCM4300, there were nine candidate polymorphisms with scores less than 5. The real SNP in the *nemR* (*ydhM*) promoter (intergenic SNP at position 1,684,163) had a score of 0. There was one cluster of putative polymorphisms with scores of 4, at position 3,386,780 (*chiA*, four lesions). This cluster was due to a sequencing error. The putative polymorphism at position 482,379 was due to an assembly error in an rhs element and the one at position 4,365,487 (*yjgB*) was due to a homopolymer error. We confirmed that this putative polymorphism was absent by direct resequencing and likewise showed in this way that predicted polymorphisms in *yhjk* and *tus* were not actually present.

For strain NCM4370, there were four candidate polymorphisms with scores less than 5. Because we had been unable to identify a candidate mutation in this strain manually, we rechecked its phenotype and found that it had not, in fact, regained faster growth at low NH_3_. Hence we believe this strain contains no new mutation. Two of the candidate lesions are at the same position, 2,093,824, and are due to a repeat region assembly error, as is the candidate lesion at position 3,194,510. The remaining candidate lesion in *ybaM* is a homopolymer error. The *tesB* and *amtB* lesions in this strain have already been discussed.

For strain NCM4384, there were five candidate lesions with false positive scores ≤2 and then the score increased to 5. The real *sroG* lesion (intergenic SNP at position 3,110,779) was among the candidate lesions with a score of 0. The new *mioC*Δ in NCM4384 did not appear in the table because precisely this same deletion was present in two other strains, NCM4299 and 4300. It had occurred during introduction of a *rutE*::kan lesion into their progenitor by transduction [3]. However, the *mioC*Δ in NCM4384 was apparent among the best candidates in the “seven strain” polymorphism table. A pair of candidates in *yibA* with a score of 2 is due to a homopolymer error. The former were assembly errors and the latter a homopolymer error. The *appA* lesion was likewise a homopolymer error and the *ppdA* lesion was a sequencing error. The *glnL* lesion in this strain has been discussed.

Finally, for strain NCM4401 there were 13 candidate lesions with false positive scores ≤2, after which the score increased to 5. A new lesion in *ntrB* (*glnL*), which has not yet been studied genetically but can reasonably account for the phenotype, was among the candidate lesions with good scores. It was detected because it was at a different position from the *ntrB* lesion present in other strains that were sent for sequencing. Likewise, the parental *amtB* lesion in NCM4384 was detected because it was different from those in NCM4287 and NCM4370. The silent lesion in *amtB* and the linked *tesB* lesion have already been discussed. Two candidate lesions with good scores were in *yebN* and were due to a homopolymer error(s), as were candidate lesions in *citG*, *speF*, *chbC* and at position 143,074. Candidate lesions at positions 656,344 and 1,006,055 and 3,585,866 were sequencing errors and those at positions 3,194,548 and 3,527,980 were repeat region assembly errors.

In summary, seven of the 10 new mutations in our seven strains with the best sequence coverage were identified in the single strain tables among the 41 total putative polymorphisms with scores <5 (Table S4). The three mutations that were missed were the two IS186 insertions in the *lon* promoter and the *mioC*Δ in strain NCM4384, which was a known mutation in two other strains. Many of the 33 putative polymorphisms that were not real mutations were readily eliminated by further sequence inspection facilitated by the tools in CoGe.

### Construction of the virtual genome of strain NCM3722

Strain NCM3722 was not sequenced; however, analysis of eight of its descendants should enable an accurate estimate of its genome sequence. To complement syntenic mapping of NCM3722 against MG1655 by SynMap and complete the virtual genome of this physiologically robust *E. coli* wild-type strain, we determined manually what caused the 62 contig breaks that were common to our four strains with the highest sequence coverage and that were flanked by unique sequence (see Materials and Methods). This number does not include contig breaks that were flanked by repetitive sequence and hence is lower than the number of contig breaks (or contigs) for individual strains given in Table 2. Of the 62 common contig breaks, more than half (33) were caused by insertion sequences (Table 6). The remainder were caused by: rRNA clusters (7); tRNA clusters (5); rhs elements (reference [18]) (5); the repeated genes *tufA/B* and *gadA/B* (4); tail fiber genes of different prophage (4); other small repeats (3); and prophage lambda (1).

**Table 6:** Physical contig breaks between unique sequences.

Contig break position relative to MG1655	Cause	Contig break position relative to MG1655	Cause
15387–16732	IS186	1648867–1649572	IS2
19796–20563	IS1	1876594–1876598	IS5 (new)^d^
164580–164596	*hrpB* // *mrcB* repeat	2064180–2065381	IS5
223465–228882	rRNA H	2066965–2068297	IS2
269765–270986	IS30	2108321–2126617	IS1 (new)^d^
273175–274373	IS5	2286941–2288135	IS5
289857–290625	IS1^a^	2481860–2481868	IS1 (new)^d^
314453–315707	IS3	2512296–2513638	IS186
348901–349197	*prpB* // *prpC* repeat	2519073–2519543	tRNA cluster (4)
380483–381815	IS2	2724093–2729049	rRNA G
390932–392187	IS3	2773185–2773189	IS2 (new)^d^
522485–526765	rhsD	2815804–2816503	tRNA cluster (5)
566003–567258	IS3	2994381–2995713	IS2
577424–580885	DLP12 *tfa* b	3128167–3129361	IS5^g^
607231–608273	IS186	3184118–3185448	IS2
695881–696280	tRNA cluster (7)	3192854–3192938	*sibDE*
728806–736085	rhsC and rhs-like^c^	3421448–3427025	rRNA D
779777–781308	tRNA cluster (7)	3468185–3469270	*tufA* f
806560	λ	3581450–3582218	IS1
916093–916101	IS1 (new)^d^	3617295–3621450	rhsB^c^
1049001–1049768	IS1	3650059–3651257	IS5
1093465–1094722	IS3	3664203–3665603	gadA^f^
1206724–1208842	e14 *tfa* b	3720206–3764339	rhsA^c^
1286289–1286845	tRNA cluster (4)	3936750–3936758	IS5 (new)
1394064–1395266	IS5	3939464–3945055	rRNA C
1409921–1433009	RAC^e^	4033187–4038859	rRNA A
1465934–1467264	IS2	4164308–4170080	rRNA B
1467306–1468540	IS30	4173967–4175151	*tufB* f
1525929–1527962	rhsE^c^	4205555–4211703	rRNA E
1568677–1569826	gadB^f^	4478826–4478833	IS1 (new)
1630940–1633863	QIN *tfa* b	4505482–4506722	IS30

a The region between two IS1 insertions in MG1655 (11 kb) has been deleted in NCM3722 and only one IS1 insertion remains.

b Tail fibers of prophage.

c Rearrangement hot spots [18], [19].

d Occurs in NCM3722 but not in MG1655.

e Contig break could also be caused by IS5 in the same location.

f This gene is repeated. The *gadA* and *gadB* genes are the same. The *tufA* and *tufB* genes are the same.

g There is an insertion of 7 kb of new sequence and an additional copy of IS5 in NCM3722.

### Genome comparison between strains NCM3722 and MG1655

There were 18 large differences between the genomes of NCM3722 and reference strain MG1655 (Table S1). To determine small genetic differences between these strains we compared a composite sequence from NCM4139, NCM4287, and NCM4370, which had the highest sequence coverage, to MG1655 computationally as described in Materials and Methods (Fig. 1). Again, we made comparison only at positions for which the sequence of the three NCM strains was invariant. The 51 small differences between NCM3722 and MG1655 detected in this way included synonymous (2), non-synonymous (33), and intergenic (9) SNPs, and frameshift mutations (7) (Table S5). The non-synonymous SNPs gave rise to both missense and nonsense changes in proteins. Many of the missense changes yielded variant proteins that had been observed in other *E. coli* lineages (e.g. the five changes in the *opp* operon; Table S5). A SNP in *glnX* created an amber suppressor that is associated with the presence of an amber mutation in *rpoS* in many *E. coli* K12 strains [21], as in NCM3722, and the frameshift mutation in *ylbE* restored an intact ancestral gene of unknown function.

The MG1655 isolate that was sequenced (now CGSC7740 and ATCC700926) carries four known lesions (*rph-1*, *ilvG*, *eut*, *rfb-50*), and the MG1655 isolate from the *E. coli* Genetic Stock Center, CGSC6300, carries an additional lesion (*fnr-267*) [4], [22]. NCM3722 is *rph* ^+^, *ilvG* ^+^, and *fnr* ^+^. Like MG1655, NCM3722 is *eut* ^−^ and therefore unable to degrade ethanolamine due to an insertion of a cryptic prophage in the *eut* operon. The insertion appears to be the same and in the same position in both strains. We previously hypothesized that the position of the insertion might be different in NCM3722 based on gene expression profiling [4]. However, that hypothesis did not take into account the requirement for the transcriptional activator EutR, which is coded for by the most distal gene in the operon. Finally, NCM3722 does not carry the IS5 insertion in *wbbL* (also called *yefJ*; b2031, b2030) that is present in MG1655 (*rfb-50* mutation; [23]), but it does carry an 18 kb deletion from the closely linked *rfbA* gene (b2039) through *wcaF* (b2054). Hence, like MG1655, NMC3722 should lack the O-antigen portion of the lipopolysaccharide. The deletion would account for loss of expression of genes *galF* (b2042) — *rfbA* (b2039) in NCM3722 relative to MG1655 because it covers these genes and it would account for decreased expression of genes *wbbI* (b2034) — *gnd*(b2029) because it covers the *rfbA* promoter [4].

Two striking differences in gene expression between NCM3722 and MG1655 were observed previously: MG1655 had higher expression of the galactitol operon, whereas NCM3722 had much higher expression of the flagellar and chemotaxis regulon [4]. Higher expression of the galactitol operon in MG1655 appears to be due to disruption of the gene for the galactitol repressor, *gatR*, only in this strain. Increased expression of the flagellar and chemotaxis regulon in NCM3722 may be accounted for by large differences in the promoter regulatory region for the master regulatory genes *flhDC* (Table S1) that increase their expression [4]. In support of this, we have observed in retrospect that two of the single genes with much higher expression in NCM3722 than MG1655 were *yhjH* (b3525), a motility gene, and *aer* (b3072), the gene for the aerotaxis receptor, both of which are known to be activated by FlhDC directly (*yhjH*) or through the flagellar sigma factor FliA (*yhjH* and *aer*) (http://biocyc.org/ecocyc/index.shtml).

Several other genetic differences between NCM3722 and MG1655 can also be associated with differences in gene expression. An insertion in *rbsR* (b3753), which inactivates the ribose repressor, apparently accounts for increased expression of the *rbs* operon (b3748–b3753) in NCM3722. A mutation in the regulatory region for *iclR* (isocitrate lyase R; b4018), which is predicted to eliminate autorepression and lead to increased *iclR* expression (Table S5), apparently accounts for reduced expression of the *aceBA* operon (b4014 and b4015) in NCM3722 [4]. The products of this operon — malate synthase and isocitrate lyase — constitute the glyoxylate bypass. Due to the absence of an IS5 insertion in the *nmpC* gene (b0552) (Table S1) NCM3722 expresses the NmpC outer membrane protein.

We considered the significance of the 7 kb of new sequence inserted in NCM3722, and which is present in many *E. coli* K12 strains (Table S1). It restores an intact *yghO* gene (b2981), which is predicted to encode a DNA-binding transcriptional regulator (http://biocyc.org/ecocyc/index.shtml), and carries genes that code for a predicted acyl-CoA synthase, an NAD-dependent epimerase dehydratase, a phosphopantetheine-binding protein, and an oxononanoate synthase. These would be transcribed divergently from *yghO*. The gene for the predicted pantetheine binding protein is interrupted by an IS5 insertion and hence would not be expressed, and the insertion might also decrease or eliminate expression of the predicted oxononanoate synthase. Adjacent to the previous four genes are two genes that encode a predicted member of the *yjgP*/*yjgQ* permease family and that would be transcribed toward the other four. The oxononanoate synthase is homologous to *bioF* and hence we investigated whether the other genes might be involved in biotin synthesis. Specifically, we asked whether or not the acyl-CoA synthase was homologous to the pimeloyl-CoA synthase, BioW, of *Bacillus sphaericus*, or YhfT and YhfS, putative fatty acid-CoA ligases associated with biotin-related operons in other bacteria by looking for a relationship to the proteins from *Sinorhizobium meliloti*. Second, we asked if the permease proteins were homologous to permease proteins CbiO and CbiQ, whose genes are often clustered with the biotin permease gene *bioY* (again using the *S. meliloti* proteins). Third, we asked if there was a binding site for the biotin regulatory protein BirA near the inserted genes [24]. We obtained no evidence supporting any of these three conjectures. Hence, we looked for other pathways in which the proteins encoded in the insertion might function by performing a BlastP search of bacterial genomes not closely related to the proteobacteria. We found that the acyl-CoA synthase has homologs in *Bacillus cereus*, *Nostoc punctiforme*, and *Streptomyces scabies*. The genes for these homologues lie in operons dedicated to polyketide synthesis. Remarkably the operon in *N. punctiforme* also includes genes for an NAD-dependent epimerase and phosphopantetheine-binding protein homologous to those in the insertion. Other homologues of acyl-CoA synthase in the NCM3722 insertion have been tentatively assigned to fatty acid metabolism.

## Discussion

New sequencing technology promises to facilitate identification of mutations in diverse species. Our example showed the feasibility of using 454 whole genome shotgun sequencing to detect a variety of types of spontaneous mutations in *E. coli* K12. Roche provided raw sequence data for eight strains and tables of differences between each strain and the non-parental reference strain MG1655 (∼350/strain), which we analyzed manually to find known mutations and identify new ones. We showed genetically that the mutations identified in four strains were necessary and sufficient for the phenotypes we had selected. Because the manual analysis was laborious and time-consuming and comparison to MG1655 produced many false positive and negative differences, we sought a better approach. We had Roche assemble contigs *de novo* rather than by using a reference genome, developed an algorithm to assemble contigs to pseudomolecules by synteny to the genome of MG1655, and wrote a custom Perl program to find polymorphisms in the aligned genomes. Among the eight strains, we originally identified 1800 putative polymorphisms that contained all of the mutations we had found manually. To reduce the number of putative polymorphisms and facilitate detection of true mutations, we considered appropriate combinations of strains and devised ranking systems to penalize known sources of error. After verifying mutations known to be present in the strains at the outset, we identified new mutations most easily by comparing the sequence of each individual strain to a composite of the others. In the seven strains with highest sequence coverage we identified seven of the 10 new mutations among the 41 total putative polymorphisms with the lowest false positive scores (<5; Table S4). Many of the 33 putative polymorphisms that were not real mutations were readily eliminated by further sequence inspection facilitated by the tools in CoGe. The three new mutations that were missed are special cases analyzed in Results.

The mutations present in our eight strains at the outset included a frameshift mutation, a small deletion, an insertion of a small drug-resistance element, and several intragenic SNPs (Table 1). Mutations acquired after selection for new phenotypes included a frameshift, a large insertion, a large deletion, and five intragenic and extragenic SNPs. Inversions are known to be rare [15], [25]. Hence our spectrum included all of the common sorts of mutations that occur in bacteria.

We found new insertion mutations in two of our strains by a laborious manual analysis focused on contig breaks that occurred in a minority of strains. We were also able to locate these insertion mutations computationally by finding new contig breaks in the two strains on syntenic dotplots to MG1655 and analyzing the sequence around the breaks using CoGe's suite of tools for comparative genomics. (Fig. 4). However, locating the mutations visually depended on the fortunate placement of new breaks at a different place from common breaks and did not yield the identity of the repetitive element that was inserted (IS186). Systematically locating insertion mutations caused by repetitive elements will require either paired end sequencing, which is more expensive, or the development of algorithms to analyze contig breaks. Identifying insertions of elements that occur once in a genome, such as the lambda prophage or the kanamycin cassette in *tesB*, is not a problem.

We identified mutations using a simple scoring system to penalize polymorphisms that were likely to be due to homopolymer sequencing errors, which are caused by 454 sequencing technology, or misassembly errors, which are caused by assembly algorithms (Table S4; http://genomevolution.org/paper_supp_data/8-Ecoli-genomes-2010/). Considering only strains with adequate sequence coverage and applying the scoring system allowed us to produce a short list of candidate mutations from ∼10^9^ nt of raw sequence data. CoGe facilitated examination of sequence around the candidate mutations, which indicated that most were likely to be due to residual homopolymer errors or misassembly errors. The residual homopolymer errors are easy to identify and misassembly errors tend to occur in clusters. To detect sequencing errors other than homopolymer errors, one must return to raw sequence data, but the candidate list can be shortened sufficiently to be tractable experimentally without having to do this (Table S4). The analysis leads quickly to mutations that can be confirmed or eliminated by resequencing and subsequently analyzed genetically to determine their bearing on phenotype. Finding different alleles in the same gene, a frequent result of selections [5], is not a problem. In our example we detected two new mutations in *nemR* and one in the *nemR* promoter.

We were unaware that one of the eight strains we sent for sequencing (NCM4370) did not have the phenotype we had selected and hence probably contained no new mutation. There were only four promising leads in this case and all were easily eliminated bioinformatically as described above. Thus, this strain served as a negative control. Using sequencing to identify mutations in two of our strains (NCM4139 and NCM4384) was particularly rewarding. Despite the fact that both were isolated spontaneously, each had acquired two mutations that contributed to its phenotype, a rare occurrence. One mutation (the *nemR* or *sroG* lesion) was responsible for the bulk of the phenotypic change — acquisition of the ability to grow on pyrimidines as sole nitrogen source at high temperature. The second mutation, an insertion of IS186 in the promoter for the *lon* gene that is characteristic of *E. coli* B strains [14], [26], [27], had no demonstrable effect by itself but yielded better growth in combination with either the *nemR* or *sroG* lesion. Moreover, these pairs of mutations were the major means of acquiring the ability to grow on pyrimidines at high temperature. Though it would have been straightforward to locate the primary *nemR* or *sroG* lesion using classical mapping techniques, it would have been difficult to find the insertion in the *lon* promoter. Given that many *E. coli* labs are no longer equipped for classical mapping and that sequencing and the requisite data analysis are becoming cheaper and easier, sequencing is becoming the method of choice [28].

The only unselected mutation we confirmed in our eight strains was an intergenic SNP (between *agaA* and *agaS*) in NCM4139, which is one of the strains selected for growth on pyrimidines at high temperature. Oddly, a previous deletion in the *E. coli* K12 lineage resulted in an N-terminal truncation of *agaA* and caused loss of the ability to grow on N-acetyl galactosamine as carbon source, the Aga^−^ phenotype [29]. Although the *mioC*Δ present in NCM4387, the other strain selected for growth on pyrimidines at high temperature, appeared to be unselected, this same deletion occurred independently in a related strain (the parent of strains NCM4299 and 4300) and hence we believe it may confer some subtle benefit (Table 1; [3]). We have not yet analyzed the *chpS* lesion in NCM4287 or the *yehD* lesion in NCM4781 genetically and hence cannot rule out that they are unselected. Our protocol after isolating and purifying new strains is to freeze them at −80°C and handle them as populations thereafter unless there is a specific reason to do otherwise. It is our understanding that unselected mutations are rarely fixed in such populations. The estimated rate of accumulation of unselected spontaneous SNPs is ≤3.6×10^−10^ per base pair per generation ([27] and references cited therein). For one SNP our frequency would be ∼2.7×10^−10^ (4.5×10^6^ bp each for seven strains and 816 generations for 34 single colony isolations in their history and the assumption that a single colony contains 10^7^ cells).

### Insights into using 454 sequencing to detect mutations in *E. coli*

This study revealed or confirmed a number of means to aid in identification of mutations by deep sequencing. As discussed in Results it is beneficial to have seven or eight related strains sequenced simultaneously rather than comparing a single mutant to a parent. This parallel sequencing of multiple strains also makes it unnecessary to have a complete genome sequence of the parental strain: one can work in any *E. coli* background. Sequence coverage of 30-fold is optimal (Fig. 2; Table S2). Although paired end sequencing, which is considerably more expensive, is not required, as discussed above it is useful for detecting insertion mutations caused by repetitive elements. Sequence coverage of <20-fold is inadequate and 50-fold coverage is not advantageous because the Newbler assembly program used by Roche apparently begins to misassemble repeat regions. To employ our methods, contigs should be assembled *de novo* and can then be ordered and oriented to create pseudo-molecules by mapping to a reference strain using the syntenic path assembly algorithm (Fig. 3) (see Materials and Methods and Results) [30]. This method will assemble inversions incorrectly if they occur in repetitive sequences that cause contig breaks. As noted above, visual display of syntenic mapping for several strains may facilitate detection of a new insertion as a new contig break (Fig. 4) and, if so, one can then use CoGe's tool GEvo for high resolution sequence comparison of the region in multiple strains to determine precisely where the insertion has occurred, although not what has caused it. Lists of putative polymorphisms can be generated by Mauve and PolyMFind (http://genomevolution.org/paper_supp_data/8-Ecoli-genomes-2010/). The bulk of the errors in these lists will be homopolymer sequencing errors due to 454 technology (Fig. 5, Table S3) and Newbler assembly errors caused by local repetitive sequences. Hence our simple scoring system for penalizing these errors should provide a useful starting point for greatly reducing the number of putative polymorphisms to be considered. Homopolymer sequencing errors will make it difficult to detect true frameshift mutations in long homopolymers (5 or greater) (Table S3), but we readily detected frameshift mutations in shorter homopolymers. Homopolymer errors would obscure mutations caused by a frameshift mutagen [31]. Assembly errors would cause a problem detecting clustered mutations induced by the mutagen N-methyl-N′-nitro-N-nitrosoguanidine [32]. Though Illumina sequencing does not give rise to homopolymer sequencing errors, the short read lengths that can be achieved at present will cause more contig breaks in shorter repetitive sequences and hence will make syntenic mapping more difficult.

### Broader applicability

The computational tools we have developed should be useful for identifying mutations in Bacteria and Archaea, as long as there is a closely related strain with a complete annotated genome. The occurrence of large numbers of insertion sequences or other repetitive elements would be uniquely problematic in de novo assembly of contigs and a very high GC or AT content would increase problems with homopolymer sequencing errors and repeat sequence assembly errors due to lower overall sequence complexity. The comparative genomics tools available in CoGe, together with its repository of over 9000 genomes, will facilitate rapid analysis of whole genomes and smaller regions. These tools will help lead quickly to putative polymorphisms that should be resequenced to confirm them and/or analyzed genetically.

### Assembly of the virtual genome of NCM3722 and comparison to MG1655

We have been able to identify all of the elements that cause contig breaks flanking unique sequence in mutant strains derived from strain NCM3722. Hence we have been able to complete a virtual assembly of its genome that should be useful to others employing NCM3722 for physiological and genetic studies. Though we could identify a number of specific genetic differences responsible for improving the growth of NCM3722 with respect to that of the sequenced strain MG1655 and a number of differences in specific regulatory proteins and promoters (e.g. *flhDC*, *gatR*, *evgA*, *malT*, *rbsR*, *iclR* and the promoter for *kgtP*), it is not clear whether these are sufficient to account for the general physiological robustness of NCM3722. Other things that may contribute include: the presence of the F plasmid; differences in the organization of the genome due to large insertions and deletions (Table S1); small differences in components of the macromolecular synthesis machinery (e.g. RpoD, RpoS, RpsG, RF-2; Table S5).

### Relationship to other work

High throughput sequencing has been used recently to do classical genetic studies in *B. subtilis* [33] and the nematode worm *C. elegans* [34]. The *B. subtilis* studies, which focused on detection of SNPs, revealed a synergistic role of *relA* suppressor mutations that arose sequentially at two different loci. Though the authors do not comment on it, there was also a remarkably high occurrence of additional mutations in the *B. subtilis relA* suppressor strains. High throughput sequencing has also been used in ground breaking studies of the genetic differences between lineages of *E. coli* [27], [35], [36], [37], *Pseudomonas syringae* [38], *Caulobacter crescentus* [39], and *B. subtilis* [33]. It has been used elegantly to follow the evolutionary progression of *E. coli* strains under constant environmental conditions in the laboratory [40], [41] and to monitor the evolution of cheating and cooperation in *M. xanthus* [42]. At this time the combination of paired end (paired read) sequencing together with new algorithms that allow paired end reads to be assembled into complete microbial genomes appears to be a method of choice for using sequencing to identify mutations [43], [44], [45]. These tools must be complemented by software that allows experimentalists to perform complex analyses of the data easily. The software and analytical workflow we developed for this project are steps in that direction. We are working toward enabling researchers to upload their raw sequence data or partially assembled genomes to CoGe and then click through tools available there to assemble and annotate their genomes and select a set of them for detection, classification, and ranking of polymorphisms. The extraordinary pace of advances in sequencing technology and computational analysis will continue to make detection of true polymorphisms easier — and cheaper — and this in turn promises to reinvigorate classical microbial genetics.

## Supporting Information

Figure S1 **High resolution analysis of a syntenic discontinuity between MG1655 and NCM4370 caused by insertion of prophage lambda into the latter.** The syntenic discontinuity is marked by a red arrow in Fig. 3. The top and middle panels represent the same genomic region from MG1655 and NCM4370, respectively. The bottom panel represents the genomic DNA from lambda phage. The dashed line in the middle of each panel separates the top and bottom strands of the genomic DNA. Genes are represented as colored arrows and are above or below the line if they are transcribed from the top or bottom strand of the DNA, respectively. Regions of homology, as identified by pair-wise blastz comparisons, are shown as colored blocks. Transparent wedges have been drawn to connect regions of homology and reveal the insertion of prophage lambda in NCM4370. The vertical orange line for NCM4370 represents the 100 Ns used to join two contigs. The analysis can be regenerated at http://genomevolution.org/r/aob
(TIFF)

Figure S2 **Genomic distribution of tRNAs in reference strain MG1655 and strain NCM4139.** The red triangles (direction arbitrary) show the genomic locations of tRNAs and the numbers indicate the presence of multiple tRNAs at a single location.
(TIFF)

Figure S3 **Screen shot of polymorphism table for the seven strains with highest sequence coverage.** The table (7-strain table at http://genomevolution.org/paper_supp_data/8-Ecoli-genomes-2010/) can be sorted by position on the genome (position) –as shown here, false positive score, type of mutation (type) or size. The table includes links to panels and tools in CoGe that show raw data aligned for multiple genomes and facilitate identification of homopolymer and misassembly errors. The repeated occrrence of *aceF* on the screen shot shown is due to a misassembly error.
(PDF)

Table S1 **Table of large differences between MG1655 and NCM3722.**
(PDF)

Table S2 **Raw polymorphisms other than contig breaks.**
(PDF)

Table S3 **Homopolymer error as a function of homopolymer length.**
(PDF)

Table S4 **Short list of putative unique mutations in seven strains**
(DOC)

Table S5 **Table of small differences between MG1655 and NCM3722**
(DOC)
