# Supplementary figures and images for: Using Genomic Sequencing for Classical Genetics in E. coli K12
Source: PLoS One. 2011 Feb 25;6(2):e16717. doi: 10.1371/journal.pone.0016717 (PMC3045373; doi:10.1371/journal.pone.0016717)

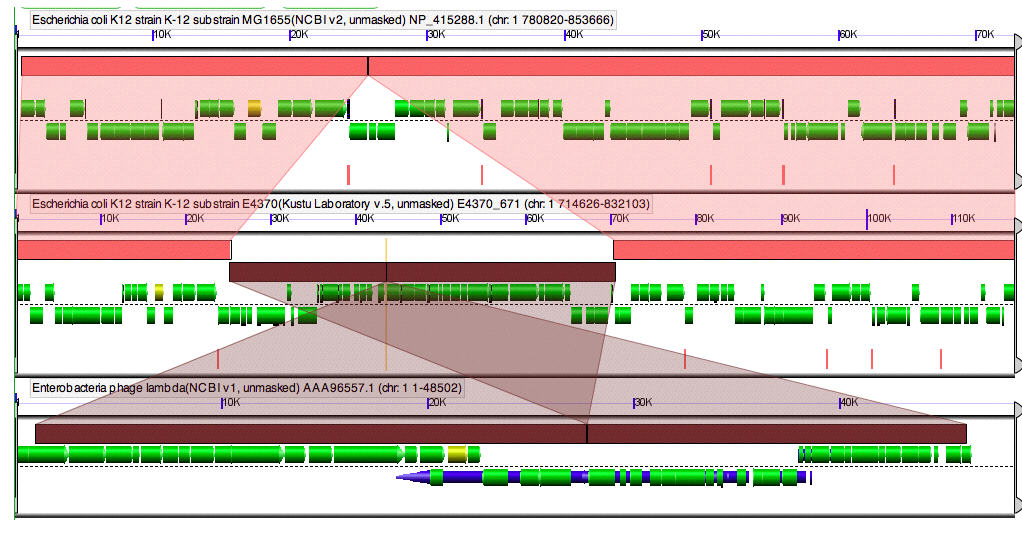

Supplement: Figure S1 — High resolution analysis of a syntenic discontinuity between MG1655 and NCM4370 caused by insertion of prophage lambda into the latter. The syntenic discontinuity is marked by a red arrow in Fig. 3. The top and middle panels represent the same genomic region from MG1655 and NCM4370, respectively. The bottom panel represents the genomic DNA from lambda phage. The dashed line in the middle of each panel separates the top and bottom strands of the genomic DNA. Genes are represented as colored arrows and are above or below the line if they are transcribed from the top or bottom strand of the DNA, respectively. Regions of homology, as identified by pair-wise blastz comparisons, are shown as colored blocks. Transparent wedges have been drawn to connect regions of homology and reveal the insertion of prophage lambda in NCM4370. The vertical orange line for NCM4370 represents the 100 Ns used to join two contigs. The analysis can be regenerated at http://genomevolution.org/r/aob (TIFF) [file pone.0016717.s001.tiff]

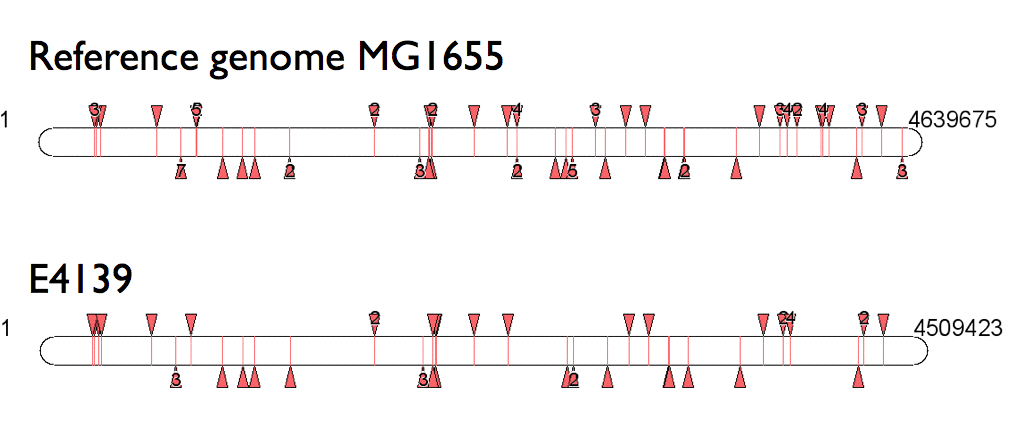

Supplement: Figure S2 — Genomic distribution of tRNAs in reference strain MG1655 and strain NCM4139. The red triangles (direction arbitrary) show the genomic locations of tRNAs and the numbers indicate the presence of multiple tRNAs at a single location. (TIFF) [file pone.0016717.s002.tiff]
