# Supplementary material for: Using Genomic Sequencing for Classical Genetics in E. coli K12
Source: PLoS One. 2011 Feb 25;6(2):e16717. doi: 10.1371/journal.pone.0016717 (PMC3045373; doi:10.1371/journal.pone.0016717)
Supplement: Figure S3 — Screen shot of polymorphism table for the seven strains with highest sequence coverage. The table (7-strain table at http://genomevolution.org/paper_supp_data/8-Ecoli-genomes-2010/) can be sorted by position on the genome (position) –as shown here, false positive score, type of mutation (type) or size. The table includes links to panels and tools in CoGe that show raw data aligned for multiple genomes and facilitate identification of homopolymer and misassembly errors. The repeated occrrence of aceF on the screen shot shown is due to a misassembly error. (PDF) [file pone.0016717.s003.pdf]

POSITION	POSITION SORT	GENE	FALSE POSITIVE SCORE	TYPE	SUBTYPE	SIZE	SIZE SORT	POLYMORPH SUMMARY	NCM4139	NCM4287	NCM4299	NCM4300	NCM4370	NCM4384	NCM4401	10_NT_ALIGN	ANNOTATION	(STRAND)CODON	GEVO_LINK	COGE_ALIGN_LINK	HOMOPOLYMER FALSE POSITIVE SCORE	MULTI-HIT FALSE POSITIVE SCORE
46,171	46171	-	0	contig join	-	-	-	-						N		show	-	-	GEvo Link<br>GEvo_Link_20k	-	0	0
48,332	48332	araB, b0063	6	insertion	Frameshift	1	1	--T----	-	-	T	-	-	-	-	show	L-ribulokinase	show	GEvo Link<br>GEvo_Link_20k	CoGeAlign Link	6	0
57,023	57023	b0070, setA	8	indel	Frameshift	1	1	TT-TTT-	T	T	-	T	T	T	-	!<br>TACCTGCTGGTTTTTTTTTTATT<br>TACCTGCTGGTTTTTTTTTTATT<br>TACCTGCTGG-TTTTTTTTTATT<br>TACCTGCTGGTTTTTTTTTTATT<br>TACCTGCTGGTTTTTTTTTTATT<br>TACCTGCTGGTTTTTTTTTTATT<br>TACCTGCTGG-TTTTTTTTTATT<br>hide	broad specificity sugar efflux system	show	GEvo Link<br>GEvo_Link_20k	CoGeAlign Link	8	0
63,009	63009		8	insertion		1	1	-----A-	-	-	-	-	-	A	-	show	INTERGENIC		GEvo Link<br>GEvo_Link_20k		8	0
63,714	63714		5	insertion		1	1	-----T-	-	-	-	-	-	T	-	show	INTERGENIC		GEvo Link<br>GEvo_Link_20k		5	0
70,349	70349	b0082, mraW	7	insertion	Frameshift	1	1	---A---	-	-	-	A	-	-	-	show	S-adenosyl-dependent methyltransferase activity on membrane-located substrates, putative apolipoprotein	show	GEvo Link<br>GEvo_Link_20k	CoGeAlign Link	7	0
105,354	105354	-	0	contig join	-	-	-	-							N	show	-	-	GEvo Link<br>GEvo_Link_20k	-	0	0
105,593	105593	aceF, b0115	31	SNP	Synonymous	1	1	CCCCCCT	C	C	C	C	C	C	T	show	pyruvate dehydrogenase (dihydrolipoyltransacetylase component), pyruvate dehydrogenase, dihydrolipoyltransacetylase component E2	show	GEvo Link<br>GEvo_Link_20k	CoGeAlign Link	0	31
105,597	105597	aceF, b0115	31	SNP	Nonsynonymous Amino acids: I V	1	1	AAAAAAG	A	A	A	A	A	A	G	show	pyruvate dehydrogenase (dihydrolipoyltransacetylase component), pyruvate dehydrogenase, dihydrolipoyltransacetylase component E2	!<br>(1):ATC CTG GTG AAA GTT<br>(1):ATC CTG GTG aAA GTT<br>(1):ATC CTG GTG AAa GTT<br>(1):GTG ATG GTG aaa GTg<br>hide	GEvo Link<br>GEvo_Link_20k	CoGeAlign Link	0	31
105,599	105599	aceF, b0115	31	SNP	Nonsynonymous Amino acids: I V	1	1	CCCCCCG	C	C	C	C	C	C	G	show	pyruvate dehydrogenase (dihydrolipoyltransacetylase component), pyruvate dehydrogenase, dihydrolipoyltransacetylase component E2	show	GEvo Link<br>GEvo_Link_20k	CoGeAlign Link	0	31
105,600	105600	aceF, b0115	31	SNP	Nonsynonymous Amino acids: L M	1	1	CCCCCCA	C	C	C	C	C	C	A	!<br>GACCGAAATCCTGGTGAAAGT<br>GACCGAAATCCTGGTGAAAGT<br>GACCGAAATCCTGGTGAAAGT<br>GACCGAAATCCTGGTGAAAGT<br>GACCGAAATCCTGGTGAAAGT<br>GACCGAAATCCTGGTGAAAGT<br>GACTGAAGTGATGGTGAAAGT<br>hide	pyruvate dehydrogenase (dihydrolipoyltransacetylase component), pyruvate dehydrogenase, dihydrolipoyltransacetylase component E2	!<br>(1):CTG GTG AAA GTT GGC<br>(1):CTG GTG aAA GTT gGC<br>(1):CTG GTG AAa GTT GGC<br>(1):ATG GTG aaa GTg ggC<br>hide	GEvo Link<br>GEvo_Link_20k	CoGeAlign Link	0	31
105,611	105611	aceF, b0115	31	SNP	Synonymous	1	1	TTTTTTG	T	T	T	T	T	T	G	show	pyruvate dehydrogenase (dihydrolipoyltransacetylase component), pyruvate dehydrogenase, dihydrolipoyltransacetylase component E2	show	GEvo Link<br>GEvo_Link_20k	CoGeAlign Link	0	31
105,682	105682	-	0	contig join	-	-	-	-			N	N		N		show	-	-	GEvo Link<br>GEvo_Link_20k	-	0	0
