# Supplementary material for: Using Genomic Sequencing for Classical Genetics in E. coli K12
Source: PLoS One. 2011 Feb 25;6(2):e16717. doi: 10.1371/journal.pone.0016717 (PMC3045373; doi:10.1371/journal.pone.0016717)
Supplement: Table S1 — Table of large differences between MG1655 and NCM3722. (PDF) [file pone.0016717.s004.pdf]

SUPPLEMENTARY TABLE 1. Table of large differences between MG1655 and NCM3722

Position	MG1655	NCM3722	Genes Affected
278387-279154<br>289858-290625	IS1<br>IS1	IS1<br>11 kb deletion<br>279155-290625	Deletion of 11 genes of cryptic prophage CP4-6 by apparent recombination of IS1 elements
573810-575013	IS5	No insert	Outer membrane porin gene *nmpC* of DLP12 prophage interrupted by IS5 in MG1655 is intact in NCM3722
687070-688272	IS5	No insert	IS5 upstream of *gltIJKL* operon (glutamate/aspartate transport) in MG1655 is not present in NCM3722
806552-806565	No insert	Lambda	NCM3722 is a lambda lysogen. Lambda phage has wild type lambda sequence
916093-916101	No insert	IS1	IS1 interrupts *ybjD* gene. YbjD is a putative ATP-dependent endonuclease of OLD family
1876598-1876594	No insert	IS5	IS5 interrupts *yeaP* gene. YeaP is a putative diguanylate cyclase
1976527-1977294<br>1977312-1977316	IS1	Tn1000	Insert sites for IS1 and Tn1000 are separated by 9 base pairs. Both are upstream of the *flhDC* operon and disrupt the regulatory region in potentially different ways. FlhDC is the principal regulator of flagellar sysnthesis and swarming
2099769-2100970	IS5	No insert	Lipopolysaccharide biosynthesis gene *wbbL* interrupted by IS5 in MG1655 is intact in NCM3722

2108321-2126617	No insert	IS1 and 18 kb deletion	Genes deleted are *rfbA, rfbD, rfbB, galF, wcaM, wcaL, wcaK, wzxC, wcaJ, cpsG, cpsB, wcaI, fcl, gmd, wcaF*. All are related to lipopolysaccharide and capsule formation.
2168198-2169452	IS3	No insert	Repressor of galactitol utilization operon *gatR* interrupted in MG1655 is intact in NCM3722
2481860-2481868	No insert	IS1	IS1 interrupts *evgA* gene. EvgA is a transcriptional activator that interacts with sensor histidine kinase EvgS to regulate acid and drug resistance
2773185-2773189	No insert	IS2	IS2 inserted between pseudogenes *yfjW* and *ypjI*, remnants of CP4-57 cryptic prophage
3128165-3129367	IS5	2 IS5 elements 7 kb sequence not in MG1655	7 kb sequence includes genes like those of *E.coli* strain BL21(DE3) at this location. The six genes code for a conserved protein, an acyl CoA synthetase, 2 permeases, an epimerase, and an oxomonoate synthase. Appears that sequence was deleted in MG1655
3363724-3364740	IS5	No insert	The *yceF* gene interrupted in MG1655 is intact in NCM3722. It lies in a cluster of genes of unknown function
3936750-3936758	No insert	IS5	IS5 inserted into *rbsR*, transcriptional repressor for ribose metabolism operon *rbsDACBKR.*
4478833-4478826	No insert	IS1	IS1 inserted in *yjgN*, which encodes an inner membrane protein of unknown function
4496204-4497534	IS2	No insert	IS2 element located between pseudogenes in KpLE2 phage

F plasmid

In addition to the above large differences, NCM3722 carries an F plasmid of 67 kb. The F plasmid is identical to the 99159 bp plasmid annotated in Genbank (AP00198) except in two regions. The *finO* gene is intact in the NCM3722 F plasmid because the IS3 element at 1-1258 is not present. (Disruption of *finO*, which codes for the transacting fertility inhibition factor, results in constitutive *tra* expression.) The 37 kb region between 4191-41089 is absent from the F plasmid in NCM3722 and is replaced with a 6.4 kb region. The 37 kb region that is missing consists of 22 genes including the *repB* gene, which codes for the RepFIB replication protein of plasmid P307. The 6.4 kb region that replaces it carries an intact *repA* gene, which codes for the RepFIC protein. (This gene is disrupted by Tn1000 in the missing 37 kb region.) It also contains 2.7 kb that codes for a PHP N-terminal protein, a putative phosphoesterase, and 1.3 kb without similarity to any sequence in the database.
