# Supplementary material for: Using Genomic Sequencing for Classical Genetics in E. coli K12
Source: PLoS One. 2011 Feb 25;6(2):e16717. doi: 10.1371/journal.pone.0016717 (PMC3045373; doi:10.1371/journal.pone.0016717)
Supplement: Table S2 — Raw polymorphisms other than contig breaks. (PDF) [file pone.0016717.s005.pdf]

SUPPLEMENTARY TABLE 2. Raw polymorphisms other than contig breaks[a]

	NCM 4139	NCM 4287	NCM 4299	NCM 4300	NCM 4370	NCM 4384	NCM 4401	NCM 4781	Eight strains	Seven strains[b]
SNPs	29	8	9	14	14	15	33	157	279	131
Synonymous	15	1	4	5	0	1	3	76	105	32
Non-Synonymous	13	4	3	6	2	3	7	35	73	37
Non-Coding SNPs	1	3	2	3	12	11	23	46	101	62
Small Insertions/Deletions[c]	23	34	30	68	26	87	123	487	876	401
Frameshift[d]	13	20	15	40	17	50	76	358	589	239
Intergenic	10	14	15	28	9	35	47	129	377	164
Large Insertions/Deletions	1	1	1	1	2	0	1	2	9	12
Total	53	43	40	83	42	100	157	646	1164	544

[a] Predicted using Mauve and PolyMFind; see Fig. 1

[b] Strain NCM 4781, which has only 10-fold coverage, was omitted. The numbers in this column are derived from a separate seven strain table, not from this eight strain table.

[c] Most of these are putative homopolymer errors: 21 for NCM4139; 31 for NCM4287; 30 for NCM4299; 67 for NCM4300; 26 for NCM4370; 86 for NCM4384; 122 for NCM4401; 426 for NCM4781.

[d] Within predicted coding sequence.
