# Supplementary material for: Using Genomic Sequencing for Classical Genetics in E. coli K12
Source: PLoS One. 2011 Feb 25;6(2):e16717. doi: 10.1371/journal.pone.0016717 (PMC3045373; doi:10.1371/journal.pone.0016717)
Supplement: Table S3 — Homopolymer error as a function of homopolymer length. (PDF) [file pone.0016717.s006.pdf]

SUPPLEMENTARY TABLE 3. Homopolymer error as a function of homopolymer length

Homopolymer length	Number of homopolymers[a]	Homopolymer errors[b]	Missing nucleotide	Extra nucleotide	Percent error[c]
1	17120976	6	0	6	.000035
2	4622230	2	1	1	.000043
3	1113493	6	2	4	.00054
4	292083	24	2	22	.0082
5	94688	45	13	32	.047
6	28216	110[d]	48	62	.39
7	7000	103[d]	67	36	1.5
8	1458	68	61	7	4.7
9	129	19	17	2	15

[a] For seven strains.

[b] Calculated as described in Materials and Methods for the seven strains with the highest sequence coverage.

[c] Calculated by dividing the number of putative errors by the total number of homopolymers of each length.

[d] Between homopolymers of length 6 and 7, the chance that a nucleotide will be erroneously missed becomes higher than the chance that one will be erroneously added.
