# Supplementary material for: Using Genomic Sequencing for Classical Genetics in E. coli K12
Source: PLoS One. 2011 Feb 25;6(2):e16717. doi: 10.1371/journal.pone.0016717 (PMC3045373; doi:10.1371/journal.pone.0016717)
Supplement: Table S4 — Short list of putative unique mutations in seven strains (DOC) [file pone.0016717.s007.doc]

SUPPLEMENTARY TABLE 4. Short list of putative unique mutations in seven strains^a^

| Strain | Position | Gene | False Positive Score | | Annotation | Source of error^b^ |
| --- | --- | --- | --- | --- | --- | --- |
| NCM4139 | **1,684,686** | **b1649, *ydhM***  **(=*nemR*)** | **0** | **predicted DNA-binding transcriptional regulator** | | **none** |
|  | 2,295,130 | b2244, *yfaD* | 0 | conserved protein | | sequencing |
|  | **3,206,230** |  | **0** | **intergenic** | | **none** |
|  | 3,989,967 | b3910, *yiiM* | 0 | conserved protein | | assembly |
| NCM4287 | 2,021,439 |  | 0 | intergenic | | homopolymer |
|  | 2,969,777 |  | 0 | intergenic | | homopolymer |
|  | **4,318,525** | **b4224, *chpS*** | **0** | **antitoxin of the ChpB-ChpS toxin-antitoxin system, suppressor of inhibitory function of ChpB, PemI-like, autoregulated** | | **none** |
|  | 4,376,124 | E4287_4085 | 2 |  | | homopolymer |
| NCM4299 | **1,684,434** | **b1649, *ydhM***  **(=*nemR*)** | **0** | **predicted DNA-binding transcriptional regulator** | | **none** |
|  | 2,920,767 |  | 0 |  | | sequencing |
| NCM4300 | 482,379 |  | 0 |  | | assembly  (rhs element) |
|  | 1,643,469 | b1610, tus | 0 | DNA-binding protein, DNA-binding protein inhibition of replication at Ter sites, inhibitor of replication at Ter, DNA-binding protein | | ?^d^ |
|  | **1,684,163** | **(*pnemR*)^c^** | **0** | **SNP** | | **none** |
|  | 3,589,426 | b3529, *yhjk* | 0 | predicted diguanylate cyclase | | ?^d^ |
|  | 4,365,487 | b4269, *yjgB* | 0 | predicted alcohol dehydrogenase, Zn-dependent and NAD(P)-binding, putative oxidoreductase | | homopolymer^d^ |
|  | 3,386,780 | b3338, *chiA* | 4 | periplasmic endochitinase | | sequencing |
| NCM4370 | 444,772 | b0466, *ybaM* | 0 | predicted protein | | homopolymer |
|  | 3,194,510 |  | 0 | intergenic | | assembly (repeat region) |
|  | 2,093,824 |  | 2 |  | | assembly (repeat region) |
| NCM4384 | 1,026,886 | *appA*, b0980 | 0 | phosphoanhydride phosphorylase, phosphoanhydride phosphorylase pH 2.5 acid phosphatase, phosphoanhydride phosphorylase pH 2.5 acid phosphatase periplasmic | | homopolymer |
|  | 2,888,964 | b2826, *ppdA* | 0 | conserved protein, prepilin peptidase dependent protein A | | sequencing |
|  | **3,110,779** | **(*sroG*)^e^** | **0** | **intergenic** | | **none** |
|  | 3,667,642 | b3594, *yibA* | 2 | lyase containing HEAT-repeat | | homopolymer |
| NCM4401 | 143,974 |  | 0 | intergenic | | homopolymer |
|  | **427,489** | ***amtB*, b0451** | **0** | **ammonium transporter, probable ammonium transporter** | | **none** |
|  | 596,974 | b0613, *citG* | 0 | triphosphoribosyl-dephospho-CoA transferase | | homopolymer |
|  | 656,344 |  | 0 | intergenic | | sequencing |
|  | 666,694 | b0693, *speF* | 0 | ornithine decarboxylase isozyme, inducible | | homopolymer |
|  | 1,006,055 |  | 0 | intergenic | | sequencing |
|  | 1,779,197 | b1737, *chbC* | 0 | N,N'-diacetylchitobiose-specific enzyme IIC component of PTS, PEP-dependent phosphotransferase enzyme II for cellobiose, arbutin, and salicin | | homopolymer |
|  | 3,194,548 |  | 0 | intergenic | | assembly (repeat region) |
|  | 3,527,980 |  | 0 | intergenic | | assembly |
|  | 3,585,866 |  | 0 | intergenic | | sequencing |
|  | **3,942,814** | **b3869, *glnL***  **(=*nemR*)** | **0** | **histidine protein kinase sensor for GlnG regulator (nitrogen regulator II, NRII), sensory histidine kinase in two-component regulatory system with GlnG** | | **none** |
|  | 1,864,437 | b1821, *yebN* | 2 | conserved inner membrane protein | | homopolymer |

^a^ Layout is taken directly from CoGe with additions given in parenthesis. Repeated occurrences at the same location have been omitted. Entries for new mutations or in one case a unique known mutation are in bold.

^b^ Assessed by manual inspection of surrounding sequence.

^c^ The lesion is in the promoter for *ydhM* = *nemR*.

^d^ Resequencing indicated the putative polymorphism was not real.

^e^ The lesion is in *sroG*, the riboswitch for *ribB.*
