# Supplementary material for: Using Genomic Sequencing for Classical Genetics in E. coli K12
Source: PLoS One. 2011 Feb 25;6(2):e16717. doi: 10.1371/journal.pone.0016717 (PMC3045373; doi:10.1371/journal.pone.0016717)
Supplement: Table S5 — Table of small differences between MG1655 and NCM3722 (DOC) [file pone.0016717.s008.doc]

SUPPLEMENTARY TABLE 5. Table of small differences between MG1655 and NCM3722

| Location | Gene | Change^a^ | Annotation | Comments |
| --- | --- | --- | --- | --- |
| 377,924 | *frmA* | A → C  Val → Gly | glutathione-dependent formaldehyde dehydrogenase | variant in BL21 |
| 547,694 | *ylbE* | A → G  synonymous |  |  |
| 547,832 | *ylbE* | ∆ → G  fs | pseudogene | restores intact ancestral gene of unknown function |
| 695,693 | *glnX* | C → T  CUG → CUA  anticodon | gln tRNA | Amber suppressor  Inserts Q |
| 902,471 | *artP* | G → T  Leu → Met | arginine transporter | variant not seen |
| 1,100,766 | *csqG* | T → A  Lys → Stop | curli production  assembly/transport component | amino acids  270 amino acids truncated to 47 |
| 1,169,059 | *ycfS* | A → G  Leu → Pro | L, D-transpeptidase linking Lpp to murein | variant in HS |
| 1,189,203 | *phoP* | A → G  synonymous | 2-component regulator with PhoS |  |
| 1,298,687 | intergenic | T → G | upstream of *oppABCDF* oligopeptide transport operon | near Lrp binding site |
| 1,300,016 | *oppA* | A → T  Asn → Tyr | periplasmic binding protein of ATP-dependent oligopeptide transporter | variant in B185 |
| 1,300,023 | *oppA* | G → A  Ser → Asn |  | variant in B185 |
| 1,300,214 | *oppA* | A → G  Asn → Asp |  | variant in B185 |
| 1,303,466 | *oppD* | T → G  Val → Gly | ATP-binding component for oligopeptide transporter | variant in B strains |
| 1,304,760 | *oppF* | T → G  Ser → Ala |  | variant in B strains |
| 1,335,418 | *acnA* | A → G  Ser → Gly | aconitate hydrase I | variant in W3110 |
| 1,356,883 | *puuP* | T → C  Tyr → Cys | putrescine importer | variant in BW2952 |
| 1,641,703 | *ydfU* | A → T  Leu → Gln | predicted protein of QIN prophage | variant in 0157 clade |
| 1,650,355 | *intQ* | T → C  Phe → Leu | QIN prophage integrase | variant in IAI39 |
| 1,706,796 | *rsxC* | 96 bp deletion | electron transport protein required for reduction of SoxR | may be assembly error  requires analysis |
| 1,779,284 | *ydiR* | T → G  Ile → Ser | electron transfer flavoprotein; FAD-binding | variant not observed in other strains |
| 1,892,863 | *pabB* | T → C  Leu → Pro | aminodeoxychorismate synthase | variant in BW2952 |
| 2,001,370 | *fliC* | G → T  Asn → Lys | flagellum structural protein | variant not found |
| 2,038,457 | *yedY* | C → A  Ala → Asp | membrane-anchored periplasmic TMAO, DMSO reductase | variant in BW2952 |
| 2,145,548 | *alkA* | C → T  Trp → Stop | DNA glycosylase | variant not found |
| 2,208,964 | *yehQ* | T → G  Stop → Glu | pseudogene | 666 amino acids as in *E.coli* 101-1 |
| 2,276,205 | *yejG* | 8 bp insert | conserved protein | variant in B185 |
| 2,663,769 | *csiE* | C → T  Q → Stop | stationary phase inducible  gene of unknown function | truncates 426 → 104 amino acids |
| 2,723,840 | intergenic | A → G | promoter region of *kgtP* (α-ketoglutarate transporter) |  |
| 2,823,651 | intergenic | ∆ → C | promoter region of *mltB* membrane-bound lytic murein transglycosylase B |  |
| 2,865,477 | *rpoS* | G → A  Gln → Stop | stationary phase sigma factor (38); RNA polymerase | variant observed |
| 2,992,397 | *pbl* | ∆ → C frameshift | pseudogene | pathogenicity island remant – extends pbL as in B185 |
| 3,033,568 | *prfB* | T → C  Thr → Ala | RF-2 peptide chain elongation release factor | variant in CFT073 |
| 3,048,974 | intergenic | ∆ → C | promoter region *gcvT* aminomethyl transferase | near GcvA binding site |
| 3,212,779 | *rpoD* | T → C  Tyr → His | RNA Polymerase  sigma 70 | variant in ATCC8739 |
| 3,386,063 | *aaeB* | T → G  Thr → Pro | p-hydroxybenzoic acid efflux system component | variant in BW2952 |
| 3,471,634 | *rpsG* | A → T  Leu → Stop | S7 of 30S ribosomal subunit component | variant in *E. coli* B strain, C-terminal 23 amino acid deletion |
| 3,488,402 | *pabA* | T → G  Arg → Arg | aminodeoxychorismate synthase, subunit II |  |
| 3,552,157 | *malT* | T → A  Trp → Arg | DNA-binding transcription factor for maltose regulon | variant not seen |
| 3,660,723 | *mdtF* | C → T  gln → Stop | RpoS-dependent multidrug transporter | regulated by EvgAS |
| 3,705,970 | intergenic | C → T | upstream of *dppA* dipeptide transporter |  |
| 3,713,804 | *bisC* | A → G  Leu → Pro | biotin sulfoxide reductase | variant not seen |
| 3,723,199 | *glyQ* | T → G  Glu → Ala | tRNA synthetase α-subunit | variant in most *E.coli* |
| 3,813,903 | *rph* | ∆ → C  228 → 238 amino acids | Ribonuclease PH | variant in most *E.coli* |
| 3,949,559 | x → *ilvG* | ∆ → AT | acetolactate synthase isozyme 2 | Restores 548 amino acid protein variant in some *E.coli* |
| 3,957,957 | intergenic | C → T | upstream of *ppiC* peptidyl-prolyl cis-trans isomerase C | 100 bp upstream of transcription start |
| 4,221,661 | intergenic | T → C | upstream of *iclR* transcriptional regulator Isocitrate lysase regulator | change is 14 bp from transcription start |
| 4,241,880 | *malF* | C → A  Gly → Cys | maltose transporter subunit | variant not seen |
| 4,340,070 | *melA* | T → A  Leu → Gln | α-galactosidase NAD-binding | variant not seen |
| 4,505,447 | intergenic | G → C | pseudogene of IS911 *insO* | Interupted by IS30 and IS600 |
| 4,508,261 | intergenic | T → G | between pseudogene *yjhV* and *fecE* | 450 bp downstream of *fecE* |
| 4,540,706 | intergenic | T → ∆ | 10 bp upstream of *fimAICDFGH* transcription start | may alter phase variation of operon by affecting invertible DNA element *fimS* |

^a^ In NCM3722.
